# Aptamers as an approach to targeted cancer therapy

**DOI:** 10.1186/s12935-024-03295-4

**Published:** 2024-03-16

**Authors:** Fatemeh Mahmoudian, Azin Ahmari, Shiva Shabani, Bahman Sadeghi, Shohreh Fahimirad, Fahimeh Fattahi

**Affiliations:** 1 Cancer Research Center, Semnan University of Medical Sciences, Semnan, Iran; 2 Clinical Research Development Unit of Ayatollah-Khansari Hospital, Arak University of Medical Sciences, Arak, Iran; 3 Department of Radiation Oncology, School of Medicine, Arak University of Medical Sciences, Arak, Iran; 4 Department of Infectious Diseases, School of Medicine, Arak University of Medical Sciences, Arak, Iran; 5 Department of Community Medicine, School of Medicine, Arak University of Medical Sciences, Arak, Iran; 6 Molecular and Medicine Research Center, Arak University of Medical Sciences, Arak, Iran; 7 Oncopathology Research Center, Iran University of Medical Sciences, Tehran, Iran

**Keywords:** Aptamer, SELEX, Cancer, Targeted therapy

## Abstract

Conventional cancer treatments can cause serious side effects because they are not specific to cancer cells and can damage healthy cells. Aptamers often are single-stranded oligonucleotides arranged in a unique architecture, allowing them to bind specifically to target sites. This feature makes them an ideal choice for targeted therapeutics. They are typically produced through the systematic evolution of ligands by exponential enrichment (SELEX) and undergo extensive pharmacological revision to modify their affinity, specificity, and therapeutic half-life. Aptamers can act as drugs themselves, directly inhibiting tumor cells. Alternatively, they can be used in targeted drug delivery systems to transport drugs directly to tumor cells, minimizing toxicity to healthy cells. In this review, we will discuss the latest and most advanced approaches to using aptamers for cancer treatment, particularly targeted therapy overcoming resistance to conventional therapies.

## Background

Cancer is a leading cause of death globally. Based on the World Health Organization's report, cancer is the first or second leading cause of death before the age of 70 in 112 out of 183 countries, ranking third or fourth in an additional 23 countries [1]. This medical condition occurs when abnormal cells multiply uncontrollably and have the potential to spread to nearby or distant tissues. As cancer progresses, these cells acquire specific traits, including increased signaling for growth, resistance to cell death, unlimited replication ability, stimulation of new blood vessel formation, and activation of invasion and metastasis [2].

Early diagnosis and the implementation of appropriate treatment modalities are essential for treating cancer patients [3]. Treatment options vary depending on the type and stage of cancer which may include surgery, chemotherapy, radiation therapy, targeted therapy, or immunotherapy [4].

Conventional cancer therapies such as chemotherapy are usually insufficient in advanced aggressive tumors. Their lack of specificity leads to a high recurrence rate and high toxicity [5]. In targeted therapy, designed drugs interfere with a targeted protein which is responsible for tumor growth [6]. On the other hand, immunotherapy takes the advantage of the patient’s immune system to destroy tumor cells [7]. Although this therapeutic approach has been promising in both hematologic and solid malignancies, intrinsic resistance of tumor cells and dose-limiting side effects cause transient responses to drugs [8, 9].

Over time, researchers have studied cancer cell behavior, immune response, and microenvironment to improve individualized cancer treatment with traditional therapies and drug delivery. Despite notable progress in cancer therapies, developing a productive treatment approach remains a considerable obstacle. Promoting cancer science by utilizing innovative therapeutic targeting and delivery methods that minimize adverse effects is essential. Continuously advancing in this direction will undoubtedly lead to better outcomes for cancer patients [9–11]. One promising avenue of investigation is the use of aptamers, a class of molecules with unique physical and chemical properties. Aptamers appear to have great potential for targeted tumor treatment and can be applied in different ways, including therapeutic aptamers, aptamer-drug conjugates (AptDC), aptamer-functionalized nanoparticles, and aptamer-mediated immunotherapy [12].

In this review, we will discuss the synthesis and selection of aptamers for targeted cancer treatment to overcome resistance and reduce side effects from conventional cancer therapy. Additionally, provides an overview of the advances and challenges in aptamer development for using aptamers in cancer treatment.

## Structure and properties of aptamers

Aptamers are small molecules that possess the remarkable potency to recognize and bind to their target with high affinity. Nucleic acid and peptide aptamers are two classifications based on their structures. The name aptamer is derived from the Latin word “aptus” (to fit) and the Greek word ‘‘meros’’, (particle) due to their ligand function [13, 14].

The flexible nature of aptamers gives them the ability to wrap around a small molecule target or fit into clefts and gaps within the surface of much larger target molecules. Aptamers have the ability to bind to a wide range of targets, including peptides, proteins, small molecules, organic compounds, metal ions, and biological targets such as viruses, bacteria, yeast, and mammalian cells. This ability is due to their unique three-dimensional folding which provides high specificity in binding. The interaction between aptamers and their targets creates strong conformational adjustments, and the binding is mediated via van der Waals forces, hydrogen bonding, electrostatic interactions, stacking of flat moieties, and shape complementarity [15, 16].

Nucleic Acid aptamers (NA-Apts) are short single-stranded (20–100 bps) DNA or RNA (ssDNA or ssRNA) oligonucleotides that are folded into 3D conformations specified by stems, bulges, loops, hairpins, triplicates, pseudoknots, kissing stem-loop complexes, or G-quadruplex constructors [17]. Based on the final application, the main goal and DNA or RNA target the most appropriate aptamers can be selected. RNA-based aptamers have a relatively flexible structure compared with DNA-based, therefore RNA-based aptamers have a broader range of target molecules. However, RNA-based aptamers are more sensitive to chemical and enzymatic degradation. Moreover, the selection of RNA aptamers is more complicated as its processing requires more enzymatic steps [18].

Peptide aptamers (P-Apts) developed after Nucleic Acid aptamers. P-Apts are polypeptides that consist of a short amino acid loop (5–20 residues), embedded in to the rigid protein structure. Due to the lower conformational entropy of the restricted peptide loop, the binding affinity of P-Apts could be as much as 1000 times higher than the free peptide [19].

Aptamers possess distinct characteristics that enable them to selectively attach to a particular target. As a result, they may present a promising alternative to antibodies for targeted cancer treatment. Despite the suitability of antibodies for numerous applications, there are certain scenarios where aptamers may prove to be a superior option [20]. They are smaller and steadier than antibodies, allowing for better transport and tissue penetration. Aptamers are delivered through a basic and reasonable process and the time required to create aptamers is comparatively brief. Not at all like antibodies, aptamers don't require animals or an immune reaction for their generation [20, 21].

Since aptamers are chemically synthesized, batch-to-batch variety can be enormously diminished allowing economical, high-accuracy large-scale generation of aptamers for clinical applications. Besides, aptamer's partiality can be balanced by optimizing their acknowledgment grouping and/or by controlling authoritative response conditions. Once chosen, the stability of the aptamers can be expanded by chemical alteration of the nucleotides as well as by changing their secondary structures. Since aptamers are chemically synthesized, chemical modifications can be presented into them at any wanted position within the nucleotide chain. In spite of the fact that antibodies can be chemically altered, site-specific adjustments are extremely troublesome [22].

Moreover, through built up solid-phase chemical synthetic strategies and site-directed chemistries, labels for detection and linkers for conjugation can be effectively embedded at wanted destinations within the oligonucleotide arrangement without compromising the binding affinity or selectivity [23]. The in vitro selection step permits aptamers to be produced against something else toxic compounds that would kill the animal in antibody generation. Moreover, aptamers are steadier at high temperature and they can be recovered effortlessly after denaturation and can be repeatedly utilized (Table 1).

**Table 1:** Aptamer advantage vs. antibodies

Features	Monoclonal Antibody	Aptamer	Aptamer Advantage
Size	~ 150–170 kDa	~ 12–30 kDa (~ 30–80 nucleotides)	Aptamers, due to their small size, can penetrate tissues and cells, exhibit superior target access and blood clearance, and have a lower tendency to be toxic and immunogenic
Stability	Susceptible to high temperatures and pH changes Require refrigeration for storage The denaturation is irreversible	Fairly stable at ambient temperature The denaturation is reversible	Aptamers possess remarkable stability and remain effective even after prolonged storage. Moreover, they are capable of being transported at room temperature without any special handling requirements. This makes them a highly convenient and practical option
Target potential	Targets must produce an immune response, minimum target size ≥ 600 Daltons	Can bind to very small targets, minimum target size ≥ 60 Daltons	Aptamers can be selected against a wide range of targets, including small molecules, toxic compounds, and non-immunogenic substances
Development Process	Require immune response and animals through in vivo production and cell culture	Chemical synthesis through SELEX process	SELEX screens large molecular diversity and requires only a few nanomoles for selection, while ensuring batch-to-batch consistency and no contamination
Modification	Typically conjugated with one type of signaling or binding molecule	Can be modified at both the 5’ and 3’ end	Aptamers can be easily modified for attachment and easy addition of functionalities during synthesis
Production time and cost	The production requires long time (~ 4–6 months) and expensive in vivo procedures	The selected aptamers are chemically synthesized (~ 1–3 months), reducing the production cost	Faster development time means faster time to market or publication

## Aptamer screen and applications bioinformatics for in-silico aptamer design

Systematic Evolution of Ligands by Exponential Enrichment (SELEX) is a popular technique that isolates single-stranded DNAs or RNAs with high affinity from a large library of random sequences, which first was developed in 1990 [24, 25]. Since then, researchers have generated numerous aptamers targeting various entities, including amino acids, proteins, small metal ions, organic molecules, bacteria, viruses, whole cells, and animals [26].

**Fig. 1:**
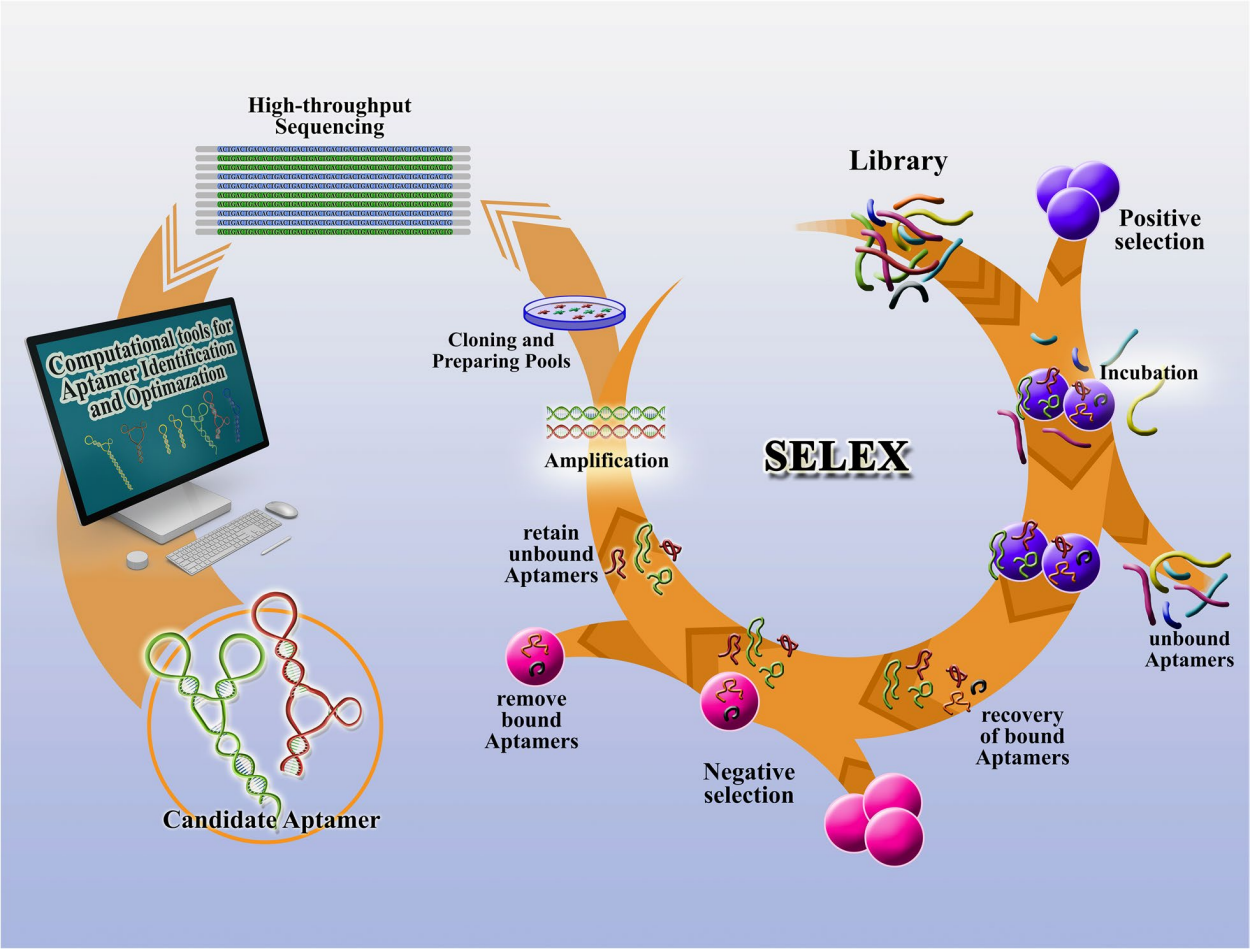
Schematic illustration of the SELEX process. Major steps involved in SELEX to identify individual aptamer

SELEX is an experimental method that determines nucleic acid aptamers capable of binding to a target molecule with high affinity and selectivity that is composed of in several steps of selection and enrichment processes [27]. First, the nucleic acid library, which consists of 10^14^–10^15^ random oligonucleotide strands, is incubated with a target molecule. Then, the target-bound oligonucleotide strands are separated from the unbound strands. The target-bound DNA or RNA strands are eluted from the target molecule and amplified via a polymerase chain reaction to seed a new pool of nucleic acids. This selection process is continued for 6–18 rounds with increasingly stringent conditions, which ensure that the nucleic acid obtained has the highest affinity to the target molecule (Fig. 1). During 6–18 rounds of selection, over 10 ^13^ different nucleic acid sequences were screened, and only a few were found to have specificity to the target. The SELEX method is versatile and can be adapted in various ways to enhance the aptamers' specificity and the SELEX process's efficiency [15]. Moreover, this method is able to produce aptamers for even unknown molecules, and this advantage has enabled the recognition of unknown surface biomarkers [28]. Cell-SELEX has been developed to screen aptamers for many types of cancer cells by using whole live cells even without the previous information of their molecular signatures [29].

Despite the different types of SELEX methods, this work is time-consuming and laborious, resulting in a low yield rate. To address these issues, the SELEX method for constructing aptamers can be combined with high-throughput sequencers. This process is commonly referred to as HT-SELEX or HTS and involves next-generation sequencing (NGS) during SELEX [27]. HT-SELEX allows screening aptamer candidates from a large number of oligonucleotide sequences within only a few days [29].

Since the inception of HT-SELEX, the use of computational bioinformatics methods for aptamer design for various targets has been gradually developed [27, 30]. Aptamer modeling and in silico design for aptamer identification and optimization may help to design better in less time [30]. Computational methods utilized for in silico aptamer identification include sequence-based, motif-searching-based, and multi-dimensional scoring-based algorithms, as well as supervised machine learning-based methods [30].

To understand the mechanisms of optimization aptamer-target interactions after obtaining aptamer candidates, aptamer structure prediction with computational methods is necessary. In this regard, 2D structures can be characterized based on their sequences, including junctions, protrusions, pseudoknots, G-quadruplexes, and triplet structures. Next, 3D structure prediction is usually done based on 2D structure. Finally, a combination of molecular docking (MD) and molecular dynamics simulation (MDS) is required to obtain an aptamer-target complex and binding sites with stability and the lowest binding energy G [27, 30, 31]. Sometimes, other methods such as the quantitative structure–activity relationship (QSAR) [32] hybrid quantum mechanics/molecular mechanics (QM/MM) studies utilized in aptamer designed [27, 33]. The Table 2 describes the prominent applications used in identification and optimization aptamer design [30, 34].

**Table 2:** Computational techniques for in silico aptamer development

Bioinformatic tool	System requirements	Description and features	Refs.
Aptamotif	Linux/Mac OS	Aptamotif is a computational method for the identification of sequence–structure RNA binding motifs in SELEX-derived aptamers	[35]
Galaxy	Web/Linux/Mac OS	Analysis of pre-processing and analyzing HTS-SELEX sequencing data using the Galaxy platform outcomes candidate aptamer sequences	[36, 37]
AptCompare	Linux/Mac OS/Windows/Galaxy	AptCompare is an automated tool designed for pre-processing and analyzing HTS-SELEX sequencing data. The tool evaluates the performance of six commonly used aptamer motif discovery programs and utilizes a meta-rank metric to identify the most promising aptamer targets	[38]
PATTERNITY.seq©	Linux/Mac OS/Windows	PATTERNITY-Seq© manages millions of sequences from raw sequencing datas to identify better aptamers in a faster way, (this tool regroups sequences in families, monitors the evolution of each family and each individual sequence, identifies enriched structure motifs, and studies the effect of selection pressure)	[39]
MEME/GLAM	Linux/Mac OS/Web	To design aptasensors by identifying motifs in aptamers through MEME analysis	[40]
MP Bind	Linux/Mac OS	MP Bind is a meta-motif-based statistical framework for predicting aptamers that bind to targets from SELEX-Seq data and proficiently managing biases caused by incomplete sequencing of aptamer pools or PCR	[41]
APTANI	Linux/Mac OS	APTANI is a computational tool to select aptamers through sequence-structure motif analysis of HT-SELEX data	[42]
APTANI^2^	Linux/Mac OS	APTANI^2^ is an expanded and optimized version of APTANI. This tool includes modules for investigating sequence motifs and secondary structures, as well as a user-friendly graphical interface and coding solutions that improve performance	[43]
AptCompare	Linux/Mac OS/Windows	AptCompare is a program that combines six analytical approaches for identifying RNA aptamer motifs across platforms	[38]
COMPAS (COMmon PAtternS)	Unknown	COMPAS (COMmon PAtternS) is a program that was developed to support the entire SELEX process can find motif combinations	[44]
RaptRankerr	Linux/Mac OS	RaptRanker as an RNA aptamer selection tool analyzes HT-SELEX data by evaluating the nucleotide sequence and secondary structure	[45]
FASTAptamer	Linux/Mac OS/Windows	FASTAptamer performs the simple tasks of counting, comparing sequences, clustering sequences, calculating fold enrichment, and searching degenerately for nucleotide sequence motifs	[46]
FASTAptameR	Web tool	FASTAptameR is an expanded set of interconnected modules such as Count, Distance, Cluster, Mutation network, Motif Discovery modules that can be used to interactively analyze and visualize HTS data	[47]
SMART-Aptamer	Mac OS/Windows	Based on multilevel structure analysis, SMART-Aptamer identifies high-affinity aptamers with low false positive and negative rates from HTS data of SELEX libraries	[48]
FSBC	Linux/Mac OS/Windows	FSBC estimates clusters considering different lengths of over-represented strings as target binding regions for HT-SELEX data	[49]
AptaPLEX	Linux/Mac OS/Windows	AptaPLEX is a utility designed specifically for demultiplexing raw HT-SELEX data into corresponding selection cycles based on barcode information	[34]
AptaSIM	Linux/Mac OS/Windows	AptaSim aimed at realistically recreating the selection process during SELEX using error-prone PCR	[50]
AptaMUT	Linux/Mac OS/Windows	A new technique has been developed to identify polymerase errors that result in improved binding affinity compared to the original sequence	[50]
AptaCLUSTER	Linux/Mac OS/Windows	AptaCluster allows for an efficient clustering of whole HT-SELEX aptamer pools	[51]
AptaTRACE	Linux/Mac OS/Windows	AptaTRACE consists of three components: data preprocessing, secondary structure profile prediction, and motif extraction. These three components are controlled through a single configuration file	[52]
AptaGUI	Linux/Mac OS/Windows	AptaGUI is a valuable resource that facilitates the implementation of the AptaTools package. This program allows for visual inspection of HT-SELEX experiments in a concise and efficient manner	[53]
AptaTools package	Linux/Mac OS/Windows	This package encompasses several algorithms, such as AptaMUT, AptaCLUSTER and AptaGUI, that are instrumental in analyzing HT-SELEX data. These algorithms enable the identification of any possible flaws in the selection protocol, the discovery of aptamer candidates, and the provision of comprehensive sequence and structure-based analysis	[53]
AptaSUITE	Linux/Mac OS/Windows	AptaSUITE incorporates a set of previously published algorithms, namely AptaPLEX, AptaSIM, AptaCLUSTER, and AptaTRACE	[54]

According to a review study, while there have been significant developments in artificial intelligence for predicting aptamer binding ability to targets, most computational tools have low citation rates. Consequently, in silico aptamer design methods have not been widely adopted [30]. Our understanding indicates that techniques like sequencing, and bioinformatic analysis are useful for aptamer screening. Additionally, technology platforms such as microfluidics, capillary electrophoresis, and flow cytometry can facilitate the isolation of aptamer candidate probes [28].

## Registered clinical trials to assess the efficacy of aptamers in the treatment of cancer

A variety of aptamer applications have been developed to target a wide spectrum of human illnesses, such as Alzheimer's disease and cancer. Despite their potential, most aptamers have not met the necessary safety and efficacy standards in human clinical trials. Various challenges hinder the widespread adoption of diagnostic and therapeutic techniques using aptamers, such as the rapid degradation, especially of RNA aptamers, by nucleases, clearance through renal filtration limiting their effectiveness, and complexities in targeting intracellular structures. Generating aptamers often requires purified target molecules, making the process time-consuming and labor-intensive. Additionally, aptamers designed to target specific molecules may also bind to structurally similar compounds, potentially leading to unintended effect [55].

For an extended period, only a single aptamer, Pegaptanib (Macugen), has demonstrated clinical efficacy. It was approved by the FDA in 2004 for treating age-related macular degeneration. Pegaptanib inhibits blood vessel development by targeting the glycosylated homodimeric VEGF isoform VEGF165. Despite its initial success, Pegaptanib was discontinued due to the emergence of more effective anti-VEGF drugs like bevacizumab, ranibizumab, and aflibercept, which are pan-blockers of VEGFAs capable of inhibiting all VEGFA isoforms. Furthermore, Pegaptanib's administration through intravitreal injections may lead to eye inflammation, pain, increased intraocular pressure, punctate keratitis, and vitreous opacity [56–58].

Recently, a second aptamer, Avacincaptad pegol (Izervay; Iveric Bio/Asetlla), brought positive news by receiving FDA approval for the treatment of geographic atrophy secondary to age-related macular degeneration in August 2023 [59].

Although there is currently no approved therapeutic application of aptamers for the treatment of cancer in a clinical setting. AS1411, a 26-nucleotide guanine-rich DNA aptamer, represented as the first aptamer to progress into clinical trials for cancer therapy. The AS1411ptamer is made up of thymine and guanine and can form guanine-mediated quadruplex structures when dissolved. The structure of AS1411 not only decreases its immunogenicity and confers resistance to nucleases but also boosts cellular uptake. By specifically targeting nucleolin, a protein that is commonly overexpressed in different tumor types, AS1411 has shown encouraging potential in combating cancer, displaying anti-proliferative effects in diverse of tumor cells through multiple signaling pathways [60, 61]. During phase I trials, three AS1411-based agents were assessed for safety and efficacy in treating advanced solid tumors and acute myeloid leukemia (AML). AS1411 was found to be non-toxic and progressed to phase II clinical trials. However, it was later discontinued from phase II trials for renal cell carcinoma (RCC) due to limited activity and low response rates in unselected patients with metastatic RC. While early indications of effective anti-cancer activity were noted in phase I and II trials for AML, further evaluation against this type of cancer has been discontinued. Nevertheless, research focusing on optimizing the structure of AS1411 remains popular [61–63].

Spiegelmers are one type of aptamer molecule that has entered clinical trials for anticancer therapeutics that a modified SELEX drug-discovery platform utilizing non-natural L-nucleotides. The L-configuration of Spiegelmers grants them resistance against degradation by nucleases present in the bloodstream, and they further exhibit low immunogenicity. These two features are crucial for nucleic acid therapeutics. Currently, NOXXON Pharma is developing Spiegelmers proficient in neutralizing chemokines within the tumor microenvironment [64].

Ongoing studies are exploring aptamer design for cancer diagnosis and treatment. For instance, NOX-E36 (emapticap pegol) is currently under investigation in clinical trials for Diabetes Mellitus and Albuminuria. The research is concentrated on their use in oncology, with preclinical data demonstrating efficacy in solid tumor models like pancreatic and liver cancer by TME Pharma (formerly NOXXON Pharma). According to Table 3, several studies registered on ClinicalTrials.gov are evaluating the use of aptamers in cancer therapy up to 2023. Given the vast number of aptamers currently being researched, additional clinical trials may be required to evaluate their effectiveness in treating cancer continuously [65].

**Table 3:** Application of aptamers in clinical trials to cancer therapy

Aptamer name	Study title	Target	Intervention/treatment	Phase	NCT number	Type of cancer
AS1411	A Study of AS1411 Combined With Cytarabine in the Treatment of Patients With Primary Refractory or Relapsed Acute Myeloid Leukemia	Nucleolin	Drug: AS1411 Drug: Cytarabine	Phase 2	NCT01034410	Acute Myeloid Leukemia
	Study of AS1411 in Advanced Solid Tumours		Drug: AS1411	Phase 1	NCT00881244	Advanced Solid Tumors
	A Phase II Study of AS1411 in Renal Cell Carcinoma		Drug: AS1411	Phase 2	NCT00740441	Metastatic Renal Cell Carcinoma
	Phase II Study of AS1411 Combined With Cytarabine to Treat Acute Myeloid Leukemia		Drug: AS1411	Phase 2	NCT00512083	Leukemia, Myeloid
EYE001	EYE001 to Treat Retinal Tumors in Patients With Von Hippel-Lindau Syndrome	VEGF	Drug: EYE001	Phase 1	NCT00056199	Hippel–Lindau disease
Olaptesed Pegol (NOX-A12)	Olaptesed (NOX-A12) Alone and in Combination With Pembrolizumab in Colorectal and Pancreatic Cancer (Keynote-559)	CXCL12/SDF-1	Drug: Olaptesed pegol—Monotherapy Drug: Olaptesed pegol + Pembrolizumab—Combination Therapy	Phase 2	NCT03168139	Metastatic Colorectal Cancer Metastatic Pancreatic Cancer
	Olaptesed With Pembrolizumab and Nanoliposomal Irinotecan or Gemcitabine/Nab-Paclitaxel in MSS Pancreatic Cancer (OPTIMUS)		Drug: Olaptesed pegol	Phase 2	NCT04901741	Metastatic Pancreatic Cancer
	NOX-A12 in Combination With Bortezomib and Dexamethasone in Relapsed Multiple Myeloma		Drug: NOX-A12	Phase 2	NCT01521533	Multiple Myeloma
	Glioblastoma Treatment With Irradiation and Olaptesed Pegol (NOX-A12) in MGMT Unmethylated Patients (GLORIA)		Drug: Olaptesed pegol Radiation: Radiotherapy Drug: Bevacizumab Drug: Pembrolizumab	Phase 2	NCT04121455	Glioblastoma
	NOX-A12 in Combination With Bendamustine and Rituximab in Relapsed Chronic Lymphocytic Leukemia (CLL)	CXCR4-CXCL12	Drug: NOX-A12	Phase 2	NCT01486797	Chronic Lymphocytic Leukemia
Lexaptepid Pegol (NOX-H94)	Efficacy of NOX-H94 on Anemia of Chronic Disease in Patients With Cancer	Hepcidin	Drug: NOX-H94	Phase 2	NCT01691040	Anemia of Chronic Disease

## Functionalizing aptamers for cancer therapy

### Aptamer-based cancer chemotherapy

Chemotherapy is still one of the main methods of cancer treatment [66]. Adequate drug delivery to tumor cells along with preservation of normal tissue is one of the factors of success and response to chemotherapy [67]. Short half-life, stimulation of immune response, non-specific delivery, and rapid distribution of chemotherapy drugs in healthy tissues may lead to side effects and severe complications. Additionally, the response to treatment can vary greatly, ranging from low to high, and the type and severity of side effects can also differ significantly [68]. Typically, the drug approach targets fast-growing cells, which can include both healthy and cancerous cells [66].

In order to improve the delivery of cytotoxic drugs, antibody–drug conjugates (ADCs) are used as anticancer drugs that can deliver drugs directly to the tumor site, thus making chemotherapy a targeted therapy. Despite 10 ADCs with approval from the FDA, but other studies indicated poorly in clinical trials [5].

Aptamer technology as a drug delivery agent has advantages over antibodies. Therefore, aptamers have been conjugated with chemotherapy drugs or other cancer treatment agents through physical or chemical. Due to the lower molecular weight of conjugation aptamer-drug conjugates (AptDC), led to faster and deeper tissue penetration, compared to ADCs has been proposed for targeted cancer therapy [5].

**Fig. 2:**
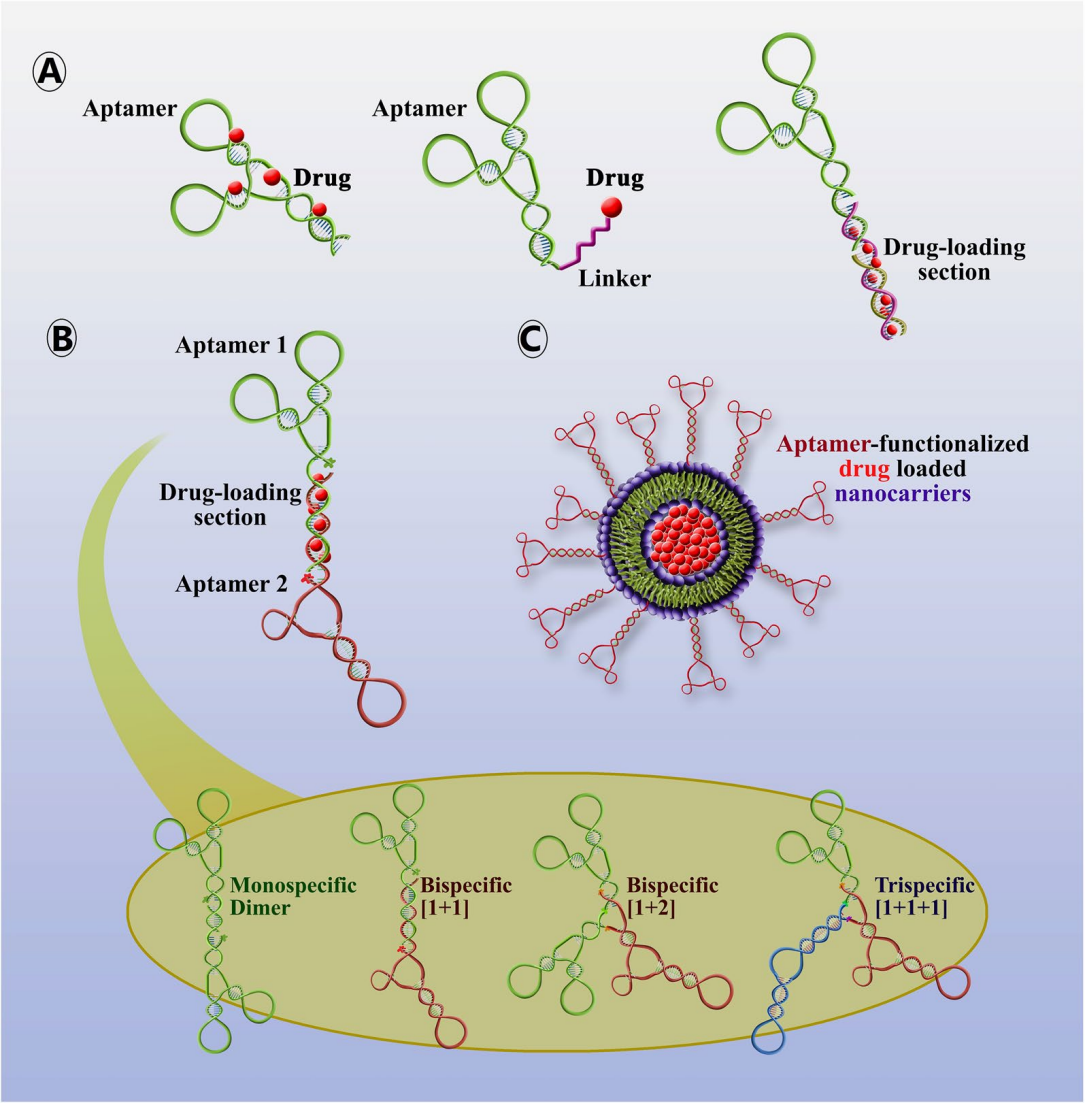
Schematic illustration of the targeted delivery of drugs using a functionalized aptamer. **A** Aptamer-drug conjugates can be created by intercalating drugs and aptamers or by using a linker. **B** Bivalent aptamers, targeting different biomarkers, linked by dsDNA to load drugs. **C** Aptamer-functionalized nanoparticles are designed for targeted drug delivery

As mentioned above, the combination of aptameric with chemotherapy drugs can be an innovative method for the selective delivery of chemotherapy agents to cancer cells in order to optimize the treatment in various studies that were investigated summarized in Table 4. Which not only increases the targeting ability but also enhances the delivery of multiple copies of drugs, by purposely engineering alterations of drug-intercalating sites on aptamers [5, 28]. Even, using this strategy may apply to highly toxic compounds that are not suitable for healthy tissues due to severe toxicity or have moderate therapeutic power, requiring a high dose of such drugs (Fig. 2A) [28].

**Table 4:** Aptamer-drug conjugations as cancer-targeted therapeutics

Aptamer names	Aptamer	Target	Drug	Type of cancer	Refs.
sgc8c	DNA	CCRF-CEM cell	Doxorubicin	T-cell acute lymphoblastic leukemia, T-cell ALL	[72]
TLS11a-GC	DNA	LH86 cell	Doxorubicin	Human hepatocellular carcinoma	[73]
MA3	DNA	MUC1 protein	Doxorubicin	Human breast, liver &lung cancer	[74]
HB5	DNA	HER2 protein	Doxorubicin	Breast cancer	[75]
EpDT3 & Scr-EpDT3	RNA	EpCAM protein	Doxorubicin	Retinoblastoma	[76]
E07 & mE07	RNA	EGFR protein	Gemcitabine 5-fluorouracil	Pancreatic cancer	[77, 78]
EpCAM aptamer	RNA	EpCAM protein	Doxorubicin	Colorectal cancer	[79]
TLS11a-GC	DNA	HepG2 cell	Doxorubicin	Hepatocellular carcinoma	[80]
AP-1-M	DNA	CD133 protein	Doxorubicin	Anaplastic thyroid cancer	[81]
AS1411/NucA	DNA	Nucleolin	Doxorubicin/Camptothecin	Breast cancer	[82]
XQ-P3	DNA	PD-L1 protein	Paclitaxel	Treating Triple-Negative Breast Cancer	[83]
AS1411	DNA	Nucleolin	Paclitaxel	Ovarian cancer	[84]
CD117-specific aptamer #1F	DNA	CD117	Methotrexate	Acute myeloid leukemia	[85]
E3	RNA	PC3 (PC-3) cells	Monomethyl auristatin E and F	Prostate cancer	[86]
XQ-2d	DNA	CD71	Monomethyl auristatin E	Uveal melanoma	[87]
P19	RNA	PDAC cell lines	Monomethyl auristatin E and derivative of maytansine 1/	Pancreatic Tumor Cell	[88]
HER2 RNA aptamer	RNA	HER2	Mertansine	Breast Cancer	[89]
S30-T1	DNA	CD33	Doxorubicin	Acute myeloid leukemia	[90]
SQ-2	RNA	ALPPL-2	5-fluoro-2′-deoxyuridine	Pancreatic ductal adenocarcinoma	[91]
APTA12 (Gemcitabine incorporated G-quadruplex aptamer)	DNA	Nucleolin	Doxorubicin/ Gemcitabine	Breast cancer	[92]
APTA12 (Gemcitabine incorporated G-quadruplex aptamer)	DNA	Nucleolin	Gemcitabine	Pancreatic cancer	[93]
G12msi aptamer	DNA	GPC3 protein	Gemcitabine	Hepatocellular carcinoma	[94]
PDGC21-T	DNA	MDA-MB-231	Gemcitabine	Triple-negative breast cancer	[95]
Modified type of AS1411	DNA	Nucleolin	Gemcitabine	Pancreatic cancer	[96]
ThioAp52	DNA	MAGE-A3	Doxorubicin	Breast, oral, pancreatic, and skin cancer	[97]

There are currently FDA-approved bispecific antibodies (bsAbs) available worldwide. These bsAbs have the unique ability to recognize two different targets. In cancer treatment, most bsAbs are developed to trigger and engage cytotoxic T cells against characteristic tumor targets present on the cancer cells. Other bsAb treatments focus on targeting cytokines immune checkpoints, and oncogenic signaling pathways [28, 69, 70]. In these regard, combining aptamers allows for simultaneous recognition of two or more different cell surface receptors (bispecific aptamers (bsApts)) or multiple copies of the same receptor (dimers of monospecific aptamers) [71]. Bivalent aptamers targeting different biomarkers, linked by dsDNA to load drugs, can produce bispecific ApDCs as a simpler, more cost-effective alternative to bivalent antibodies [28] (Fig. 2B).

### Aptamer-based cancer radiotherapy

Radiotherapy, also referred to as radiation therapy (RT), is a common treatment method for primary non-metastasis solid tumors. More than half of all cancer patients benefit from RT annually. This therapy employs high-energy radiation to shrink tumors, destroy cancer cells, and alter the microenvironment in clinics [28, 98, 99].

One of the challenges in RT for cancer patients is the insufficient dose of radiotherapy at the tumor site, which cannot be tolerated by normal tissue and may cause the risk of normal tissue damage in the treatment area. This limits the amount of radiation dose that can be administered [98–100]. Despite the obstacles, the utilization of radiosensitizers and smart targeting is increasingly attracting attention because of their capacity to specifically boost radiation effects on cancer cells at the tumor location. This strategy overcomes radio resistance and minimizes side effects, thereby presenting a prospective resolution to this dilemma [98, 99, 101].

Radiosensitizers can be specifically delivered to tumor sites by conjugating them with antibodies or aptamers, thereby ensuring their selective uptake by cancerous cells [101, 102]. Due to their exceptional specificity for the target and the wide range of previously mentioned properties, aptamers are highly adaptable and may be efficient in overcoming radio resistance. Several radiosensitizers, such as metal formulations, siRNAs, and nucleoside analogs, can be coupled with aptamers for targeted delivery into cancer cells to sensitize radiotherapy [103]. Based on information studies, metal (nano) formulations of the AS1411 and anti-MUC1 aptamers could potentially act as radiosensitizers in cancer treatment. This approach increases the levels of free radicals in tumor cells, thereby causing enforced DNA damage. In breast tumor-bearing mice, a gold nanocluster conjugate of the AS1411 aptamer demonstrated enhanced efficacy of radiation therapy. Furthermore, the anti-MUC1 aptamer was linked with the radiosensitizer 1,10 phenanthroline for in-vitro radiosensitization of breast cancer cells [103–105].

In addition to the mentioned strategy, aptamers can sensitize radiotherapy by binding to designated targets and interfering with radioresistance signaling, without any further conjugation. In this regard, a study conducted on glioblastoma has uncovered that the application of U2 aptamer, a DNA-aptamer that targets EGFRvIII, has the potential to restrain the growth, migration, and invasion of GBM cells. Moreover, it has been observed that the U2 treatment may enhance the radiosensitivity of EGFRvIII-expressing U87 cells. The researchers have speculated that the U2 treatment can hinder the DNA damage response and consequently boost the radiosensitivity in GBM cells [103, 106]. In another study, a 2'-F-RNA aptamer GL44 was used as a boron delivery agent for Boron Neutron Capture Therapy (BNCT) to target human glioblastoma U-87 malignant glioma cells, resulting in reduced tumor cell viability [107].

### Aptamer-based cancer immunotherapy

The aberrant growth, abnormal expression of membrane proteins, and escape from immune surveillance are the major hallmarks of cancer. Currently, several therapeutic approaches have been developed for cancer treatment based on our information about the interactions between the immune system and tumor cells. In spite of the intrinsic immune system being well educated to generate specific antibodies but is weakened and dysfunctional in most of the cancer patients. Therefore, to overcome this shortcoming, the application of new immunotherapeutic strategies and immune-stimulating agents for instance the increment of cancer antigenicity as well as the usage of immune modulators, cytokines, or lymphocytes are indispensable to relapsed or refractory cancer treatment [108–110]. Therefore, cancer immunotherapy offers a range of treatments, including adoptive cell therapies, cancer vaccines, immunostimulatory cytokines, oncolytic virus therapies, and antibody therapies [111].

In 1997, FDA approved the use of rituximab, a mouse-human chimeric monoclonal antibody targeting the B-cell lineage marker CD20, as a treatment for malignancy. This marked the first time a monoclonal antibody had been approved for cancer immunotherapy. Since then, over a dozen monoclonal antibodies have been approved to treat a variety of cancers [112, 113].

Despite the potential of monoclonal antibody-based therapy, it is often hindered by its high manufacturing costs and the risk of immune-related adverse effects. In contrast, nucleic acid aptamers are a stable and non-immunogenic alternative class of high affinity reagents that can be easily produced through solid-phase synthesis [114, 115].

**Fig. 3:**
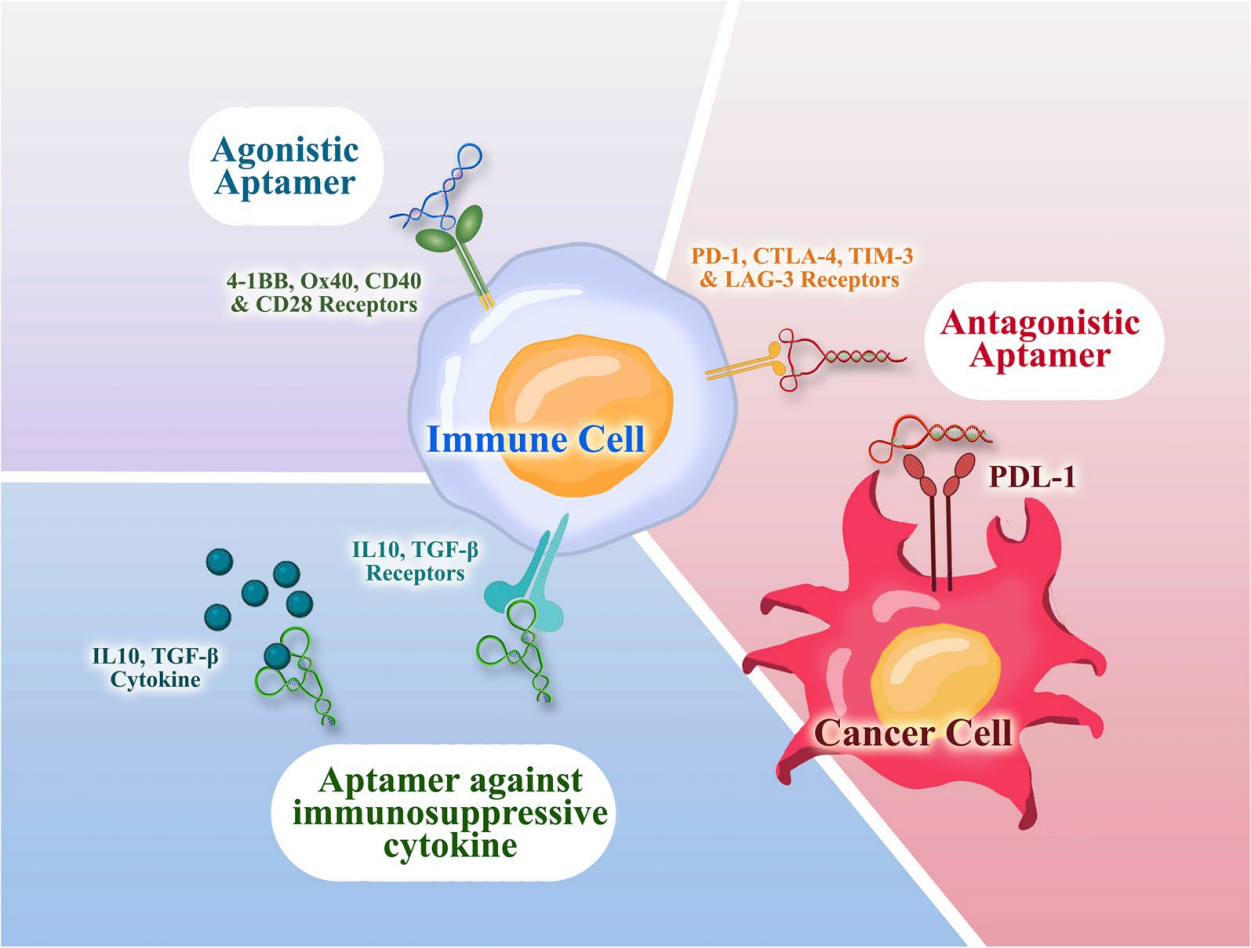
Schematic representation of immunotherapeutic aptamers for targeted cancer therapy. The immunotherapeutic aptamers are classified based on different targets, including immune checkpoint antagonists, immune receptor agonists, and inhibitors of immunosuppressive cytokines

Nowadays, aptamers with high affinity and specificity are a very promising construct as immune-modulatory agents widely used directly in therapeutic applications and drug delivery system. The immunotherapeutic aptamers belong to three major groups according to their different targets, which are immune-checkpoint antagonists, immune receptor agonists with immunostimulatory function, and inhibitors of immunosuppressive cytokines (Table 5) (Fig. 3) [116–118].

**Table 5:** Immunotherapeutic aptamers

Aptamer name	Type	Target	RNA or DNA	Modification	Function	Refs.
RNA Aptamer	Antagonistic	CTLA-4	RNA	–	Inhibition of CTLA-4 functions and enhance tumor immunity in cell and animal models	[124]
aptCTLA-4	Antagonistic	CTLA-4	DNA	–	Promotes lymphocyte proliferation, and inhibits tumor growth in both in vitro and in vivo	[125]
CTLA4apt–STAT3 siRNA	Antagonistic	CTLA-4	RNA	Covalently linked to a STAT-3 siRNA	STAT-3 gene silencing in CD8+ infiltrated lymphocytes and regulatory T cells (Tregs) in tumors and subsequently inhibits tumor growth and metastasis	[126]
MP7	Antagonistic	PD-1	DNA	Conjugated to the polyethylene glycol (PEG)	Inhibits the suppression of IL-2 secretion in primary T-cells and suppresses the tumor growth	[127]
aptPD-L1	Antagonistic	PD-L1	DNA	–	Stimulate lymphocyte proliferation in vitro, suppress tumor growth in vivo, and increase in the levels of infiltrating CD4+ and CD8+ T cells, as well as the cytokines IL-2, TNF-α, IFN-γ and the C-X-C motif chemokines	[128]
BSA-Apt	Antagonistic	PD-L1	DNA	Conjugated to the bovine serum albumin	Stronger antitumor efficacy	[129]
TIM-3 aptamer	Antagonistic	TIM-3	DNA	Trimeric form of the TIM-3 aptamer	Reduced cell death, and enhanced survival, proliferation, and cytokine secretion in vitro	[130]
LAG3 aptamer	Antagonistic	LAG3	RNA	2′-fluoro-pyrimidines	Enhances the threshold of T-cell activation	[131]
4-1BB aptamer	Agonistic	4-1BB	RNA	Bivalent and multivalent	Stimulate CD8+ T cells and inhibit tumor growth	[132]
4-1BB bispecific aptamer	Agonistic	4-1BB	RNA	2′-fluoro-pyrimidine, conjugated with vascular endothelial growth factor or osteopontin	Enhances the therapeutic index of tumor immunotherapy	[133]
hOX40 aptamer	Agonistic	OX40	RNA	2′-fluoro-pyrimidine and multimerized	Induce proliferation and IFN-γ production in activated human T cells in vitro	[134]
CD40 agonist aptamers	Agonistic	CD40	RNA	2ʹ-fluoropyrimidine	Hastened the improvement of bone marrow aplasia by inducing the reproduction and activation of B cells	[135]
CD40-blockade aptamer	Antagonist	CD40	RNA	2ʹ-fluoropyrimidine	Have a direct antitumor effect on CD40-expressing B-cell lymphoma in vitro and in vivo	[135]
CD40 agonistic aptamer-shRNA chimera	Agonistic	CD40	RNA	2ʹ-fluoropyrimidine, and linked with a short hairpin RNA targeting serine/threonine protein kinase	Enhancing tumor antigenicity by NMD inhibition and improvement in tumor infiltration and overall survival in vivo	[135]
CD28Apt2 and CD28Apt7	Agonistic	CD28	RNA	2ʹ-fluoropyrimidine and dimerization	Increasing the cellular immune system response and prolong the survival of mice	[136]
X-polymers	Antagonistic and Agonistic	CTLA-4 and CD28	RNA	CAR-like multivalent aptamer nanoparticles	Increase T cell proliferation and reverse the inhibitory effect of IL-2, and suppress the growth of mouse melanoma B16 cells both in vitro and in vivo	[137]
APT-β1	Inhibitors of immunosuppressive cytokines	TGF-β1	RNA	Combined with gefitinib	Enhanced the anti-tumor effect, and inhibit the regrowth of lung cancer	[138]
R5A1 aptamer	Inhibitors of immunosuppressive cytokines	IL-10 receptor	RNA	–	Inhibit CT26 tumor growth in mice	[123]

The classic immune checkpoint receptors CTLA-4, PD-1, PD-L1, TIM-3, and LAG-3, as well as novel ones, form a complex system of controlling the immune system, which is disrupted in cancer. The antagonistic aptamers inhibit or block the interaction of these immune checkpoints molecule with its ligand that can dysregulate the downstream signaling. Currently, these category of aptamers commonly used in clinical trials and therapeutic purposes [18, 115].

In other hand, the efficient co-stimulation of antigen-presenting cells or T cells, which is induced through the binding of a co-stimulatory receptor and its ligand, plays a crucial role in boosting antitumor immunity. Engineering multimeric versions of several RNA aptamers targeting immune co-stimulatory receptors (4-1BB, OX40, CD40 and CD28) has enabled them to act as receptor agonists to improving cancer immunotherapy [18, 119]. The majority of co-stimulatory receptors expressed in leukocytes require crosslinking of their intracellular domains in order to initiate the activation signal. This necessitates the close proximity of the receptors in order to induce the activation signal. Founded on that concept, the first agonistic aptamer was constructed as a dimer [120].

Exploring strategies to counteract immune suppression may be a productive approach to immune therapy. Tumor-infiltrating lymphocytes in the tumor microenvironment produce multiple cytokines with immunosuppressive properties. Therefore, numerous studies have developed inhibitory aptamers to neutralize these cytokines with the goal of boosting the immune system's response and enhancing tumor cell elimination [121]. TGF-β is a multifaceted cytokine with a variety of immunosuppressive effects. These effects include the suppression of T-cell proliferation, hindering the T-cell stimulatory activities of antigen-presenting cells (APCs), and impeding T-cell differentiation into helper T cells and cytotoxic T lymphocytes (CTLs) [122]. Interleukin-10 (IL-10) is another prominent immunosuppressive and anti-inflammatory cytokine that is a key mediator of immune regulation secreted in the tumor microenvironment [123].

## Aptamer-functionalized nanoparticles in drug delivery systems

Nanoscale drugs and drug delivery systems at the nanoscale level have opened up a new path for enhancing the therapeutic effectiveness of various agents and bioactive molecules by leveraging the Enhanced Permeability and Retention (EPR) effect. This effect allows for molecules or particles of a specific size to accumulate in cancer tissues more than in normal tissues [12, 139]. In other hands, nanocarriers covers a wide range of chemical combinations and protect drugs from degradation that led to increase half-life, augment cytotoxic drug payload, reduce renal clearance, control anticancer drug release kinetics, and improve solubility. A variety of therapeutic agents and biological imaging agents can be carried by nanocarriers, allowing for greater cytotoxic drug payload [140]. The use of nano-based technologies for imaging, diagnostics, and radiation therapy has increased in clinical settings [141–143].

Aptamers' in vivo applications are limited due to their vulnerability to nuclease degradation and fast renal excretion. To overcome these limitations, several attempts have been made to modify aptamers to enhance their binding affinity with the target, improve their stability, and prevent degradation by in vivo nucleases [142, 144]. In addition to adjusting the SELEX protocol, nanocarriers can improve and modify aptamers for biological applications and enhance their stability and pharmacokinetics in vivo [142].

**Fig. 4:**
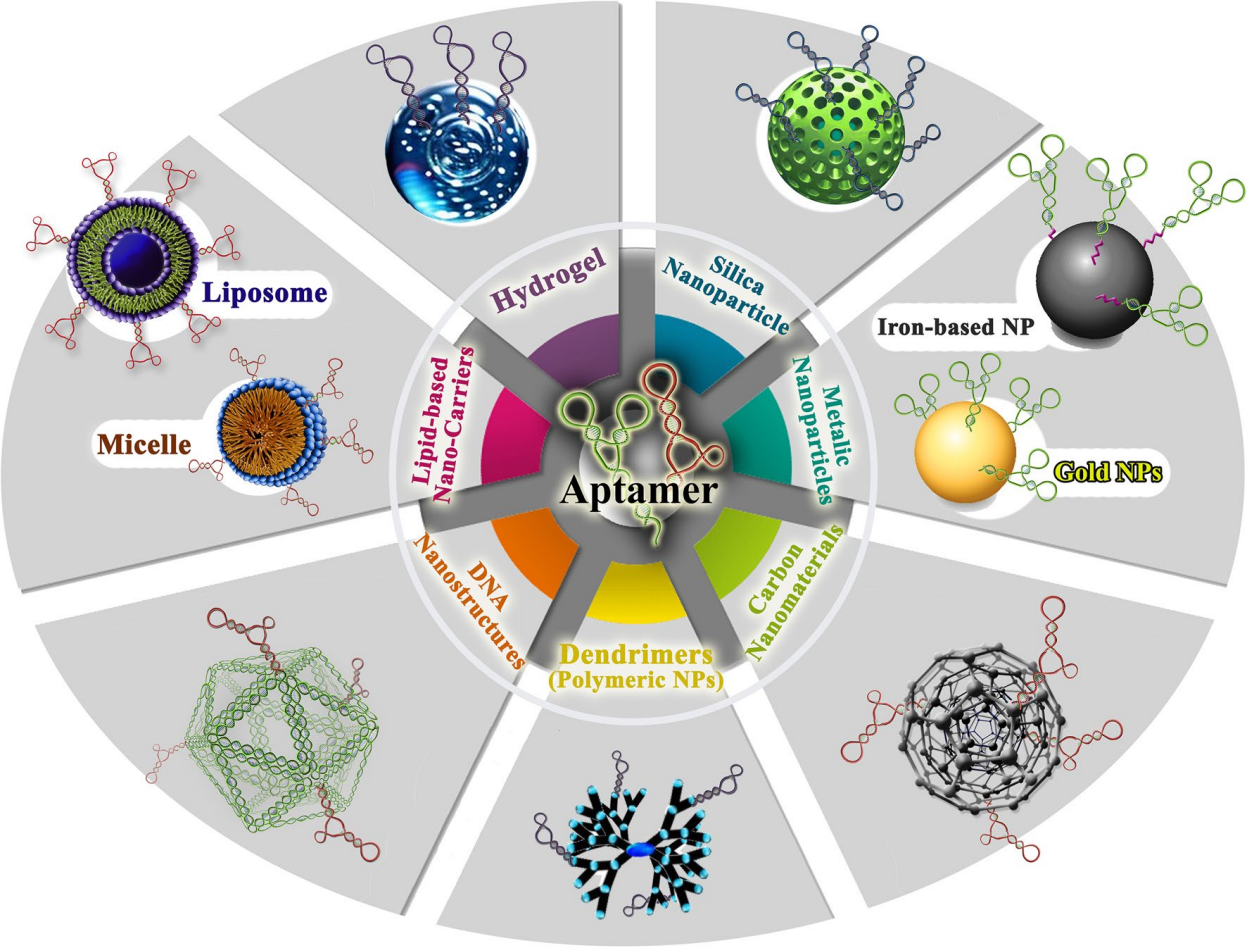
The common types of aptamer-nanocarrier conjugates for targeted drug delivery. The various types of aptamer-nanocarrier conjugates used for targeted drug delivery have been extensively explored, and are based on nanoplatforms such as liposomes, DNA/RNA origami/nanostructures, and inorganic gold or silicon nanomaterials

Combining aptamers with nanocarriers and nanoparticles can significantly enhance drug delivery efficiency (Table 6) (Fig. 2C). Nanocarriers have a high drug-loading capacity and can deliver drugs passively to specific areas, taking advantage of the enhanced EPR effect in tumors and inflamed tissues. Towards this end, aptamer-nanocarrier conjugates have been extensively explored for targeted drug delivery, based on nanoplatforms such as liposomes, DNA/RNA origamis /nanostructures, and inorganic gold or silicon nanomaterials [28] (Fig. 4). There are primarily two methods to assemble DNA/RNA nanostructures. The first method is creating "DNA tiles" by using short synthetic DNA strands, which is similar to the functioning of Lego bricks. The second method is called "DNA origami" which is a bottom-up assembly process that involves folding a long single-stranded DNA scaffold of a few thousand nucleotides and using hundreds of short staple strands to create complex 2D and 3D structures [145, 146]. Moreover, radiosensitizers-aptamers conjugated with nanoparticles radiotherapy efficiency is increased [142].

**Table 6:** Aptamer-drug conjugations with nanocarriers/nanoparticles as cancer-targeted therapeutics

Chemotherapy					
Aptamer names	Type of aptamer	Drug	Nanocarriers/nanoparticles	Type of cancer	Refs.
MUC1(5TR1)	DNA	5-fluorouracil	Chitosan carbon quantum dot	Breast Cancer	[147]
		Epirubicin	Poly (lactic-co-glycolic acid)	Breast Cancer	[148]
		Epirubicin	Super paramagnetic iron oxide nanoparticles	Colon cancer	[149]
		Doxorubicin	PEGylated liposome	Colon carcinoma	[150]
		SN-38	Chitosan nanoparticles	Colon cancer	[151]
		SN-38	Camptothecin, conjugated to hyaluronic acid	Colon cancer	[152]
		5-fluorouracil	Hyaluronan/chitosan nanoparticles	Colorectal adenocarcinoma	[153]
		Paclitaxel	Chitosan-coated human serum albumin nanoparticles	Breast cancer	[154]
5TR1 and NAS-24	DNA	Epirubicin	Selenium nanoparticles	Breast and colon cancer	[155]
MUC1 and ATP aptamer	DNA	Epirubicin	DNA diamond nanostructure	Colon carcinoma and breast cancer	[156]
AS1411		Methotrexate	Chitosan-gold nanocluster	Lung cancer	[157]
		Doxorubicin	Fe3O4@UiO-66-NH2	Breast cancer	[158]
		Doxorubicin	Polyamid-amin dendrimer grafted persistent luminescence		[159]
		5-fluorouracil	Carboxymethyl chitosan	Breast cancer	[160]
		Epigallocatechin gallate	Chitosan-silica nanoparticles	Ovarian cancer cell lines	[161]
		Erlotinib	Chitosan nanoparticles	Non-small cell lung cancer	[162]
		5-fluorouracil	Hyaluronic acid sodium salt and alginic acid sodium salt	Skin cancer	[163]
		Paclitaxel	Human serum albumin	Breast cancer	[164]
		Docetaxel	Albumin	Colon Cancer	[165]
		Doxorubicin	Albumin nanoparticles loaded on iron oxide and gold nanoparticles	Breast cancer	[166]
		Ferrocene, and purpurin	Bovine serum albumin	Breast cancer	[167]
		Doxorubicin	Bovine serum albumin	Breast cancer	[168]
AS1411 and FOXM1 Apt	DNA	Doxorubicin	Chitosan (CS)-Gold nanoparticles (AuNPs)	Lung cancer	[169]
HPA aptamers (S1.5)	DNA	Paclitaxel	PEGylated PLGA nanoparticles	Triple-negative breast cancer	[170]
Anti-PSMA	RNA	Doxorubicin	Thermally cross-linked superparamagnetic iron oxide nanoparticles (TCL-SPIONs)	Prostate cancer	[171]
Sgc8c-aptamer	DNA	Doxorubicin	N-heterocyclic carbene (NHC)–gold(I) complexe	Leukemia	[172]
Anti-EpCAM aptamer	DNA	Doxorubicin	Mesoporous silica nanoparticles	Colon cancer	[173]

Radiotherapy					
Aptamer names	Type of aptamer	Drug	Nanocarriers/nanoparticles	Type of cancer	Refs.
AS1411	DNA	-	Gold nanoclusters using bovine serum albumin capping agent	Breast Cancer	[104]
		Verapamil	Bovine serum albumin (BSA) coated silver nanoparticles (AgNPs)	Glioma	[174]
GMT8	DNA	-	PEGylated Ag@Au core–shell nanoparticles (GSGNPs)	Malignant glioma	[175]

Immunotherapy					
Aptamer names	Type of aptamer	Drug/others ingredient	Nanocarriers/nanoparticles	Type of cancer	Refs.
AS1411	DNA	CRISPR/Cas9 plasmid	Hyaluronic acid	Non-small cell lung cancer	[176]
		α-PD1 (engineered monoclonal antibodies against PD1)	PEG on nanomicelles	Breast cancer and hepatocellular carcinoma	[177]
Anti-CD16 and anti-MUC1	DNA	–	Amphipathic nanoparticles	adenocarcinomas (lung cancer and breast cancer)	[178]
PD-L1 aptamer	DNA	Fexofenadine (FEXO)	Albumin nanoparticles	Colon cancer	[179]
PD-L1 aptamer and AS1411	DNA	–	Prussian blue nanoparticles (PBs) coated with platelet membrane (PM)	Breast cancer	[180]
sTN145	RNA	PD-L1 siRNA	PLGA-based polymeric nanoparticles	Triple-negative breast cancer	[181]
APDL1(PD-L1 aptamer)	DNA	–	Gold nanorods	Non-small cell lung cancer	[182]
CTLA-4 aptamer	DNA	Fexofenadine (FEXO)	Albumin nanoparticle	Colon cancer and breast cancer	[183]
IL-4Rα aptamer	RNA	CpG oligodeoxynucleotide (ODN)	Liposome	Colon carcinoma	[184]
Endoglin aptamer (ENG-Apt)	DNA	Interferon-inducible protein-10 (IP-10)	Liposome -based nanocapsules	Melanoma tumor	[185]

## Conclusions

Cancer is a serious global threat to humanity, and medical researchers worldwide are prioritizing cancer research and therapy. The main challenge for cancer therapy is to deliver drugs to the tumor site in a way that minimizes toxicity to healthy organs. Aptamers are remarkable ligands that recognize and selectively bind to specific targets with unique properties that distinguish them from antibodies, making them ideal alternatives. They possess physicochemical properties that can be easily altered, allowing them to be functionalized with various types of drugs, such as chemotherapy, radiotherapy, and immunotherapy agents, as well as siRNA, polymers, and nanoparticles, to overcome the limitations of cancer treatment. Although aptamers have certain drawbacks such as low pharmacokinetic profile, rapid filtration and distribution to tissues from the plasma, and high susceptibility to nucleases-mediated degradation, they are increasingly attractive for use in tumor-targeted therapies. With modern biotechnology and improved aptamer screening, they can now be designed for each tumor and individual, paving the way for more precise and personalized treatments. It is important to note that this novel approach is still in its infancy, and many parameters require careful investigation. Recently, aptamers have shown promise in pre-clinical settings. We hope that clinical studies will lead to the development of therapeutic drugs for use in future medicine.

## Data Availability

Not applicable.

## Abbreviations

SELEX: Systematic evolution of ligands by exponential enrichment
FDA: Food and Drug Administration
AMD: Age-related macular degeneration
PD-1: Programmed cell death protein 1
PD-L1: Programmed cell death ligand 1
CTLA-4: Cytotoxic T lymphocyte-associated antigen-4
CAR-T: Chimeric antigen receptor-T
NGS: Next-generation sequencing
2D: 2 Dimensional
3D: 3 Dimensional
MD: Molecular docking
MDS: Molecular dynamics simulation
QSAR: Quantitative structure–activity relationship
VEGF: Vascular endothelial growth factor
CLL: Chronic Lymphocytic Leukemia
MUC1: Mucin 1
HER2: Human epidermal growth factor receptor 2
EGFR: Epidermal growth factor receptor
PDAC: Pancreatic ductal adenocarcinoma
GPC3: Glypican 3
BNCT: Boron Neutron Capture Therapy
TGF-β: Transforming growth factor beta
APCs: Antigen-presenting cells
CTLs: Cytotoxic T lymphocytes
IL-10: Interleukin-10
TIM-3: T-cell immunoglobulin mucin 3
LAG3: Lymphocyte-activation gene 3
IFN-γ: Interferon-gamma
NMD: Nonsense-mediated mRNA decay
STAT-3: Signal transducer and activator of transcription 3
Tregs: Regulatory T cells
IL-2: Interleukin-2
TNF-α: Tumor necrosis factor alpha
EPR: Enhanced Permeability and Retention
AuNPs: Gold nanoparticles
BSA: Bovine serum albumin
AgNPs: Silver nanoparticles
ODN: Oligodeoxynucleotide
PEG: Polyethylene glycol

## References

[1] Sung H, Ferlay J, Siegel RL, Laversanne M, Soerjomataram I, Jemal A (2021). Global cancer statistics 2020: GLOBOCAN estimates of incidence and mortality worldwide for 36 cancers in 185 countries. CA Cancer J Clin.

[2] Soldevilla MM, Villanueva H, Pastor F (2016). Aptamers: a feasible technology in cancer immunotherapy. J Immunol Res.

[3] Feng J, Li B, Ying J, Pan W, Liu C, Luo T (2020). Liquid biopsy: application in early diagnosis and monitoring of cancer. Small Struct..

[4] Wang JJ, Lei KF, Han F (2018). Tumor microenvironment: recent advances in various cancer treatments. Eur Rev Med Pharmacol Sci.

[5] He S, Du Y, Tao H, Duan H (2023). Advances in aptamer-mediated targeted delivery system for cancer treatment. Int J Biol Macromol.

[6] Wu H-C, Chang D-K, Huang C-T (2006). Targeted therapy for cancer. J Cancer Mol.

[7] Bashash D, Zandi Z, Kashani B, Pourbagheri-Sigaroodi A, Salari S, Ghaffari SH (2022). Resistance to immunotherapy in human malignancies: mechanisms, research progresses, challenges, and opportunities. J Cell Physiol.

[8] Ellis LM, Hicklin DJ (2009). Resistance to targeted therapies: refining anticancer therapy in the era of molecular oncology. Clin Cancer Res.

[9] Yadav P, Ambudkar SV, Rajendra PN (2022). Emerging nanotechnology-based therapeutics to combat multidrug-resistant cancer. J Nanobiotechnol.

[10] Lorscheider M, Gaudin A, Nakhle J, Veiman KL, Richard J, Chassaing C (2021). Challenges and opportunities in the delivery of cancer therapeutics: update on recent progress. Ther Deliv.

[11] Hegde PS, Chen DS (2020). Top 10 challenges in cancer immunotherapy. Immunity.

[12] Han J, Gao L, Wang J, Wang J (2020). Application and development of aptamer in cancer: from clinical diagnosis to cancer therapy. J Cancer.

[13] Eriksson ESE, Joshi L, Billeter M, Eriksson LA (2014). De novo tertiary structure prediction using RNA123—benchmarking and application to Macugen. J Mol Model.

[14] Mehta J, Van Dorst B, Rouah-Martin E, Herrebout W, Scippo M-L, Blust R (2011). In vitro selection and characterization of DNA aptamers recognizing chloramphenicol. J Biotechnol.

[15] Kong HY, Byun J (2013). Nucleic acid aptamers: new methods for selection, stabilization, and application in biomedical science. Biomol Ther.

[16] Hayashi T, Oshima H, Mashima T, Nagata T, Katahira M, Kinoshita M (2014). Binding of an RNA aptamer and a partial peptide of a prion protein: crucial importance of water entropy in molecular recognition. Nucleic Acids Res.

[17] Yang LF, Ling M, Kacherovsky N, Pun SH (2023). Aptamers 101: aptamer discovery and in vitro applications in biosensors and separations. Chem Sci.

[18] Zhou J, Rossi J (2017). Aptamers as targeted therapeutics: current potential and challenges. Nat Rev Drug Discov.

[19] Reverdatto S, Burz DS, Shekhtman A (2015). Peptide aptamers: development and applications. Curr Top Med Chem.

[20] Thiviyanathan V, Gorenstein DG (2012). Aptamers and the next generation of diagnostic reagents. Proteomics Clin Appl.

[21] Nimjee SM, Rusconi CP, Sullenger BA (2005). Aptamers: an emerging class of therapeutics. Annu Rev Med.

[22] Constantinou A, Chen C, Deonarain M (2010). Modulating the pharmacokinetics of therapeutic antibodies. Biotech Lett.

[23] Sharifi J, Khawli L, Hornick J, Epstein A (1998). Improving monoclonal antibody pharmacokinetics via chemical modification. Q J Nucl Med Mol Imaging.

[24] Stoltenburg R, Reinemann C, Strehlitz B (2007). SELEX—A (r) evolutionary method to generate high-affinity nucleic acid ligands. Biomol Eng.

[25] Liu Q, Zhang W, Chen S, Zhuang Z, Zhang Y, Jiang L (2020). SELEX tool: a novel and convenient gel-based diffusion method for monitoring of aptamer-target binding. J Biol Eng.

[26] Zhuo Z, Yu Y, Wang M, Li J, Zhang Z, Liu J (2017). Recent advances in SELEX technology and aptamer applications in biomedicine. Int J Mol Sci.

[27] Buglak AA, Samokhvalov AV, Zherdev AV, Dzantiev BB (2020). Methods and applications of in silico aptamer design and modeling. Int J Mol Sci.

[28] Zhu G, Chen X (2018). Aptamer-based targeted therapy. Adv Drug Deliv Rev.

[29] Li W, Bing T, Wang R, Jin S, Shangguan D, Chen H (2022). Cell-SELEX-based selection of ssDNA aptamers for specifically targeting BRAF V600E-mutated melanoma. Analyst.

[30] Sun D, Sun M, Zhang J, Lin X, Zhang Y, Lin F (2022). Computational tools for aptamer identification and optimization. TrAC, Trends Anal Chem.

[31] Zhang N, Chen Z, Liu D, Jiang H, Zhang Z-K, Lu A (2021). Structural biology for the molecular insight between aptamers and target proteins. Int J Mol Sci.

[32] Musafia B, Oren-Banaroya R, Noiman S (2014). Designing anti-influenza aptamers: novel quantitative structure activity relationship approach gives insights into aptamer-virus interaction. PLoS ONE.

[33] Li X, Chung LW, Li G (2016). Multiscale simulations on spectral tuning and the photoisomerization mechanism in fluorescent RNA spinach. J Chem Theory Comput.

[34] Hoinka J, Przytycka T (2016). AptaPLEX – a dedicated, multithreaded demultiplexer for HT-SELEX data. Methods.

[35] Hoinka J, Zotenko E, Friedman A, Sauna ZE, Przytycka TM (2012). Identification of sequence–structure RNA binding motifs for SELEX-derived aptamers. Bioinformatics.

[36] Thiel WH, Giangrande PH (2016). Analyzing HT-SELEX data with the Galaxy Project tools – a web based bioinformatics platform for biomedical research. Methods.

[37] Thiel WH (2016). Galaxy workflows for web-based bioinformatics analysis of aptamer high-throughput sequencing data. Mol Ther Nucleic Acids..

[38] Shieh KR, Kratschmer C, Maier KE, Greally JM, Levy M, Golden A (2020). AptCompare: optimized de novo motif discovery of RNA aptamers via HTS-SELEX. Bioinformatics.

[39] https://ris.utwente.nl/ws/portalfiles/portal/133445816/Book_of_Abstracts_Aptamers_in_Bordeaux_2019_Open.pdf.

[40] Takayama A, Medina A, Pecic S, Mohapatra A, editors. Identification of Motifs in Aptamers Using MEME Analysis to aid design of Aptasensors. 2023 IEEE 13th Annual Computing and Communication Workshop and Conference (CCWC); 2023 8–11 March 2023.

[41] Jiang P, Meyer S, Hou Z, Propson NE, Soh HT, Thomson JA (2014). MPBind: a meta-motif-based statistical framework and pipeline to predict binding potential of SELEX-derived aptamers. Bioinformatics.

[42] Caroli J, Taccioli C, De La Fuente A, Serafini P, Bicciato S (2016). APTANI: a computational tool to select aptamers through sequence-structure motif analysis of HT-SELEX data. Bioinformatics.

[43] Caroli J, Forcato M, Bicciato S (2020). APTANI2: update of aptamer selection through sequence-structure analysis. Bioinformatics.

[44] Hamada M (2018). In silico approaches to RNA aptamer design. Biochimie.

[45] Ishida R, Adachi T, Yokota A, Yoshihara H, Aoki K, Nakamura Y (2020). RaptRanker: in silico RNA aptamer selection from HT-SELEX experiment based on local sequence and structure information. Nucleic Acids Res.

[46] Alam KK, Chang JL, Burke DH (2015). FASTAptamer: a bioinformatic toolkit for high-throughput sequence analysis of combinatorial selections. Mol Ther Nucleic Acids.

[47] Kramer ST, Gruenke PR, Alam KK, Xu D, Burke DH (2022). FASTAptameR 2.0: a web tool for combinatorial sequence selections. Mol Ther Nucleic Acids..

[48] Song J, Zheng Y, Huang M, Wu L, Wang W, Zhu Z (2019). A sequential multidimensional analysis algorithm for aptamer identification based on structure analysis and machine learning. Anal Chem.

[49] Kato S, Ono T, Minagawa H, Horii K, Shiratori I, Waga I (2020). FSBC: fast string-based clustering for HT-SELEX data. BMC Bioinform.

[50] Hoinka J, Berezhnoy A, Dao P, Sauna ZE, Gilboa E, Przytycka TM (2015). Large scale analysis of the mutational landscape in HT-SELEX improves aptamer discovery. Nucleic Acids Res.

[51] Hoinka J, Berezhnoy A, Sauna ZE, Gilboa E, Przytycka TM, editors. Aptacluster–a method to cluster ht-selex aptamer pools and lessons from its application. Research in Computational Molecular Biology: 18th Annual International Conference, RECOMB 2014, Pittsburgh, PA, USA, April 2–5, 2014, Proceedings 18; 2014: Springer. 10.1007/978-3-319-05269-4_9 PMC4281958 25558474

[52] Dao P, Hoinka J, Takahashi M, Zhou J, Ho M, Wang Y (2016). AptaTRACE elucidates RNA sequence-structure motifs from selection trends in HT-SELEX experiments. Cell Syst.

[53] Hoinka J, Dao P, Przytycka TM (2015). AptaGUI—a graphical user interface for the efficient analysis of HT-SELEX data. Mol Ther Nucleic Acids..

[54] Hoinka J, Backofen R, Przytycka TM (2018). AptaSUITE: a full-featured bioinformatics framework for the comprehensive analysis of aptamers from HT-SELEX experiments. Mol Ther Nucleic Acids.

[55] Lakhin AV, Tarantul VZ, Gening LV (2013). Aptamers: problems, solutions and prospects. Acta Naturae.

[56] Fabre M, Mateo L, Lamaa D, Baillif S, Pagès G, Demange L (2022). Recent advances in age-related macular degeneration therapies. Molecules.

[57] Wieleba I, Wojas-Krawczyk K, Krawczyk P (2020). Aptamers in non-small cell lung cancer treatment. Molecules.

[58] Moon B-H, Kim Y, Kim S-Y (2023). Twenty years of anti-vascular endothelial growth factor therapeutics in neovascular age-related macular degeneration treatment. Int J Mol Sci.

[59] Shughoury A, Sevgi DD, Ciulla TA (2023). The complement system: a novel therapeutic target for age-related macular degeneration. Expert Opin Pharmacother.

[60] Gao F, Yin J, Chen Y, Guo C, Hu H, Su J (2022). Recent advances in aptamer-based targeted drug delivery systems for cancer therapy. Front Bioeng Biotechnol.

[61] Brown A, Brill J, Amini R, Nurmi C, Li Y. Development of better aptamers: structured library approaches, selection methods, and chemical modifications. Angewandte Chemie International Edition. 2024:e202318665. 10.1002/anie.202318665 38253971

[62] Thongchot S, Aksonnam K, Thuwajit P, Yenchitsomanus P-T, Thuwajit C (2023). Nucleolin-based targeting strategies in cancer treatment: focus on cancer immunotherapy. Int J Mol Med.

[63] Tong X, Ga L, Ai J, Wang Y (2022). Progress in cancer drug delivery based on AS1411 oriented nanomaterials. J Nanobiotechnol.

[64] Shigdar S, Schrand B, Giangrande PH, de Franciscis V (2021). Aptamers: cutting edge of cancer therapies. Mol Ther.

[65] Ng EW, Shima DT, Calias P, Cunningham ET, Guyer DR, Adamis AP (2006). Pegaptanib, a targeted anti-VEGF aptamer for ocular vascular disease. Nat Rev Drug Discovery.

[66] MacDonald V (2009). Chemotherapy: managing side effects and safe handling. Can Vet J.

[67] Xiang D, Shigdar S, Qiao G, Wang T, Kouzani AZ, Zhou SF (2015). Nucleic acid aptamer-guided cancer therapeutics and diagnostics: the next generation of cancer medicine. Theranostics.

[68] Kroschinsky F, Stölzel F, von Bonin S, Beutel G, Kochanek M, Kiehl M (2017). New drugs, new toxicities: severe side effects of modern targeted and immunotherapy of cancer and their management. Crit Care.

[69] Esfandiari A, Cassidy S, Webster RM (2022). Bispecific antibodies in oncology. Nat Rev Drug Discov.

[70] Reichert JM (2011). Bispecific antibodies and ADCs: once and future kings?. MAbs.

[71] Thomas BJ, Porciani D, Burke DH (2022). Cancer immunomodulation using bispecific aptamers. Mol Ther Nucleic Acids.

[72] Huang YF, Shangguan D, Liu H, Phillips JA, Zhang X, Chen Y (2009). Molecular assembly of an aptamer–drug conjugate for targeted drug delivery to tumor cells. ChemBioChem.

[73] Meng L, Yang L, Zhao X, Zhang L, Zhu H, Liu C (2012). Targeted delivery of chemotherapy agents using a liver cancer-specific aptamer. PLoS ONE.

[74] Hu Y, Duan J, Zhan Q, Wang F, Lu X, Yang X-D (2012). Novel MUC1 aptamer selectively delivers cytotoxic agent to cancer cells in vitro. PLoS ONE.

[75] Liu Z, Duan J-H, Song Y-M, Ma J, Wang F-D, Lu X (2012). Novel HER2 aptamer selectively delivers cytotoxic drug to HER2-positive breast cancer cells in vitro. J Transl Med.

[76] Subramanian N, Raghunathan V, Kanwar JR, Kanwar RK, Elchuri SV, Khetan V (2012). Target-specific delivery of doxorubicin to retinoblastoma using epithelial cell adhesion molecule aptamer. Mol Vis.

[77] Ray P, Cheek MA, Sharaf ML, Li N, Ellington AD, Sullenger BA (2012). Aptamer-mediated delivery of chemotherapy to pancreatic cancer cells. Nucleic Acid Ther.

[78] Mahajan UM, Li Q, Alnatsha A, Maas J, Orth M, Maier SH (2021). Tumor-specific delivery of 5-fluorouracil–incorporated epidermal growth factor receptor–targeted aptamers as an efficient treatment in pancreatic ductal adenocarcinoma models. Gastroenterology.

[79] Xiang D, Shigdar S, Bean AG, Bruce M, Yang W, Mathesh M (2017). Transforming doxorubicin into a cancer stem cell killer via EpCAM aptamer-mediated delivery. Theranostics.

[80] Deng R, Qu H, Liang L, Zhang J, Zhang B, Huang D (2017). Tracing the therapeutic process of targeted aptamer/drug conjugate on cancer cells by surface-enhanced Raman scattering spectroscopy. Anal Chem.

[81] Ge MH, Zhu XH, Shao YM, Wang C, Huang P, Wang Y (2021). Synthesis and characterization of CD133 targeted aptamer-drug conjugates for precision therapy of anaplastic thyroid cancer. Biomater Sci.

[82] Pusuluri A, Krishnan V, Lensch V, Sarode A, Bunyan E, Vogus DR (2019). Treating tumors at low drug doses using an aptamer-peptide synergistic drug conjugate. Angew Chem Int Ed Engl.

[83] Wu X, Li F, Li Y, Yu Y, Liang C, Zhang B (2020). A PD-L1 aptamer selected by loss-gain cell-SELEX conjugated with paclitaxel for treating triple-negative breast cancer. Med Sci Monit.

[84] Li F, Lu J, Liu J, Liang C, Wang M, Wang L (2017). A water-soluble nucleolin aptamer-paclitaxel conjugate for tumor-specific targeting in ovarian cancer. Nat Commun.

[85] Zhao N, Pei S-N, Qi J, Zeng Z, Iyer SP, Lin P (2015). Oligonucleotide aptamer-drug conjugates for targeted therapy of acute myeloid leukemia. Biomaterials.

[86] Powell Gray B, Kelly L, Ahrens DP, Barry AP, Kratschmer C, Levy M (2018). Tunable cytotoxic aptamer–drug conjugates for the treatment of prostate cancer. Proc Natl Acad Sci.

[87] Zhang H, Jin C, Zhang L, Peng B, Zhang Y, Liu Y (2022). CD71-specific aptamer conjugated with monomethyl Auristatin E for the treatment of uveal melanoma. ACS Appl Mater Interfaces.

[88] Yoon S, Huang K-W, Reebye V, Spalding D, Przytycka TM, Wang Y (2017). Aptamer-drug conjugates of active metabolites of nucleoside analogs and cytotoxic agents inhibit pancreatic tumor cell growth. Mol Ther Nucleic Acids.

[89] Jeong HY, Kim H, Lee M, Hong J, Lee JH, Kim J (2020). Development of HER2-specific aptamer-drug conjugate for breast cancer therapy. Int J Mol Sci.

[90] Yang C, Wang Y, Ge MH, Fu YJ, Hao R, Islam K (2019). Rapid identification of specific DNA aptamers precisely targeting CD33 positive leukemia cells through a paired cell-based approach. Biomater Sci.

[91] Dua P, Kim S, Lee D-K (2015). Alppl2 aptamer-mediated targeted delivery of 5-fluoro-2′-deoxyuridine to pancreatic cancer. Nucleic Acid Ther.

[92] Joshi M, Choi J-S, Park J-W, Doh K-O (2019). Combination of doxorubicin with gemcitabine-incorporated GQuadruplex aptamer showed synergistic and selective anticancer effect in breast cancer cells. J Microbiol Biotechnol.

[93] Park JY, Cho YL, Chae JR, Moon SH, Cho WG, Choi YJ (2018). Gemcitabine-incorporated G-Quadruplex aptamer for targeted drug delivery into pancreas cancer. Mol Ther Nucleic Acids.

[94] Park JY, Chae JR, Cho YL, Kim Y, Lee D, Lee JK (2020). Targeted therapy of hepatocellular carcinoma using gemcitabine-incorporated GPC3 Aptamer. Pharmaceutics.

[95] Qi J, Zeng Z, Chen Z, Nipper C, Liu X, Wan Q (2022). Aptamer-gemcitabine conjugates with enzymatically cleavable linker for targeted delivery and intracellular drug release in cancer cells. Pharmaceuticals.

[96] Hong SS, Lee S, Lee SH, Kim S, Kim D, Park H (2022). Anticancer effect of locally applicable aptamer-conjugated gemcitabine-loaded atelocollagen patch in pancreatic cancer patient–derived xenograft models. Cancer Sci.

[97] Wang C-Y, Lin B-L, Chen C-H (2021). Targeted drug delivery using an aptamer against shared tumor-specific peptide antigen of MAGE-A3. Cancer Biol Ther.

[98] Yazdian-Robati R, Bayat P, Oroojalian F, Zargari M, Ramezani M, Taghdisi SM (2020). Therapeutic applications of AS1411 aptamer, an update review. Int J Biol Macromol.

[99] Ghahremani F, Shahbazi-Gahrouei D, Kefayat A, Motaghi H, Mehrgardi MA, Javanmard SH (2018). AS1411 aptamer conjugated gold nanoclusters as a targeted radiosensitizer for megavoltage radiation therapy of 4T1 breast cancer cells. RSC Adv.

[100] Wei M, Shen X, Fan X, Li J, Bai J (2023). PD-L1 aptamer-functionalized degradable hafnium oxide nanoparticles for near infrared-II diagnostic imaging and radiosensitization. Front Bioeng Biotechnol..

[101] Safarzadeh Kozani P, Safarzadeh Kozani P, Rahbarizadeh F (2021). Flexible aptamer-based nucleolin-targeting cancer treatment modalities: a focus on immunotherapy, radiotherapy, and phototherapy. Trends Med Sci.

[102] Borbas KE, Ferreira CSM, Perkins A, Bruce JI, Missailidis S (2007). Design and synthesis of mono- and multimeric targeted radiopharmaceuticals based on novel cyclen ligands coupled to anti-MUC1 aptamers for the diagnostic imaging and targeted radiotherapy of cancer. Bioconjug Chem.

[103] Li Q, Maier SH, Li P, Peterhansl J, Belka C, Mayerle J (2020). Aptamers: a novel targeted theranostic platform for pancreatic ductal adenocarcinoma. Radiat Oncol.

[104] Ghahremani F, Kefayat A, Shahbazi-Gahrouei D, Motaghi H, Mehrgardi MA, Haghjooy-Javanmard S (2018). AS1411 aptamer-targeted gold nanoclusters effect on the enhancement of radiation therapy efficacy in breast tumor-bearing mice. Nanomedicine.

[105] Alves LN, Missailidis S, Lage CA, De Almeida CEB (2019). Anti-muc1 aptamer as carrier tool of the potential radiosensitizer 1, 10 phenanthroline in mcf-7 breast cancer cells. Anticancer Res.

[106] Zhang X, Peng L, Liang Z, Kou Z, Chen Y, Shi G (2018). Effects of aptamer to U87-EGFRvIII cells on the proliferation, radiosensitivity, and radiotherapy of glioblastoma cells. Mol Ther Nucleic Acids.

[107] Vorobyeva MA, Dymova MA, Novopashina DS, Kuligina EV, Timoshenko VV, Kolesnikov IA (2021). Tumor cell-specific 2'-Fluoro RNA aptamer conjugated with closo-dodecaborate as a potential agent for boron neutron capture therapy. Int J Mol Sci.

[108] Maimaitiyiming Y, Hong DF, Yang C, Naranmandura H (2019). Novel insights into the role of aptamers in the fight against cancer. J Cancer Res Clin Oncol.

[109] Liu W, De La Torre IG, Gutiérrez-Rivera MC, Wang B, Liu Y, Dai L (2015). Detection of autoantibodies to multiple tumor-associated antigens (TAAs) in the immunodiagnosis of breast cancer. Tumor Biol.

[110] Lee JW, Kim HJ, Heo K (2015). Therapeutic aptamers: developmental potential as anticancer drugs. BMB Rep.

[111] Oiseth SJ, Aziz MS (2017). Cancer immunotherapy: a brief review of the history, possibilities, and challenges ahead. J Cancer Metastasis Treat.

[112] Ribatti D (2014). From the discovery of monoclonal antibodies to their therapeutic application: an historical reappraisal. Immunol Lett.

[113] Abbott M, Ustoyev Y (2019). Cancer and the immune system: the history and background of immunotherapy. Semin Oncol Nurs.

[114] Li X, Li Z, Yu H (2020). Selection of threose nucleic acid aptamers to block PD-1/PD-L1 interaction for cancer immunotherapy. Chem Commun.

[115] Panigaj M, Johnson MB, Ke W, McMillan J, Goncharova EA, Chandler M (2019). Aptamers as modular components of therapeutic nucleic acid nanotechnology. ACS Nano.

[116] Haßel S, Mayer G (2019). Aptamers as therapeutic agents: has the initial euphoria subsided?. Mol Diagn Ther.

[117] Soldevilla M, Villanueva H, Pastor F (2016). Aptamers: a feasible technology in cancer immunotherapy. J Immunol Res.

[118] Kumar Kulabhusan P, Hussain B, Yüce M (2020). Current perspectives on aptamers as diagnostic tools and therapeutic agents. Pharmaceutics.

[119] Gilboa E, McNamara J, Pastor F (2013). Use of oligonucleotide aptamer ligands to modulate the function of immune receptors. Clin Cancer Res.

[120] Pastor F (2016). Aptamers: a new technological platform in cancer immunotherapy. Pharmaceuticals.

[121] Khedri M, Rafatpanah H, Abnous K, Ramezani P, Ramezani M (2015). Cancer immunotherapy via nucleic acid aptamers. Int Immunopharmacol.

[122] Gorelik L, Flavell RA (2001). Immune-mediated eradication of tumors through the blockade of transforming growth factor-β signaling in T cells. Nat Med.

[123] Berezhnoy A, Stewart CA, Mcnamara JO, Thiel W, Giangrande P, Trinchieri G (2012). Isolation and optimization of murine IL-10 receptor blocking oligonucleotide aptamers using high-throughput sequencing. Mol Ther.

[124] Santulli-Marotto S, Nair SK, Rusconi C, Sullenger B, Gilboa E (2003). Multivalent RNA aptamers that inhibit CTLA-4 and enhance tumor immunity. Can Res.

[125] Huang B-T, Lai W-Y, Chang Y-C, Wang J-W, Yeh S-D, Lin EP-Y (2017). A CTLA-4 antagonizing DNA aptamer with antitumor effect. Mol Ther Nucleic Acids..

[126] Herrmann A, Priceman SJ, Kujawski M, Xin H, Cherryholmes GA, Zhang W (2014). CTLA4 aptamer delivers STAT3 siRNA to tumor-associated and malignant T cells. J Clin Investig.

[127] Prodeus A, Abdul-Wahid A, Fischer NW, Huang EH, Cydzik M, Gariépy J (2015). Targeting the PD-1/PD-L1 immune evasion axis with DNA aptamers as a novel therapeutic strategy for the treatment of disseminated cancers. Mol Ther Nucleic Acids.

[128] Lai W-Y, Huang B-T, Wang J-W, Lin P-Y, Yang P-C (2016). A novel PD-L1-targeting antagonistic DNA aptamer with antitumor effects. Mol Ther Nucleic Acids.

[129] An Y, Li X, Yao F, Duan J, Yang X-D (2022). Novel complex of PD-L1 aptamer and albumin enhances antitumor efficacy in vivo. Molecules.

[130] Gefen T, Castro I, Muharemagic D, Puplampu-Dove Y, Patel S, Gilboa E (2017). A TIM-3 oligonucleotide aptamer enhances T cell functions and potentiates tumor immunity in mice. Mol Ther.

[131] Soldevilla MM, Hervas S, Villanueva H, Lozano T, Rabal O, Oyarzabal J (2017). Identification of LAG3 high affinity aptamers by HT-SELEX and conserved motif accumulation (CMA). PLoS ONE.

[132] McNamara JO, Kolonias D, Pastor F, Mittler RS, Chen L, Giangrande PH (2008). Multivalent 4–1BB binding aptamers costimulate CD8+ T cells and inhibit tumor growth in mice. J Clin Investig.

[133] Schrand B, Berezhnoy A, Brenneman R, Williams A, Levay A, Kong L-Y (2014). Targeting 4–1BB costimulation to the tumor stroma with bispecific aptamer conjugates enhances the therapeutic index of tumor immunotherapy stroma-targeted 4–1BB costimulation. Cancer Immunol Res.

[134] Pratico ED, Sullenger BA, Nair SK (2013). Identification and characterization of an agonistic aptamer against the T cell costimulatory receptor, OX40. Nucleic Acid Ther.

[135] Soldevilla MM, Villanueva H, Bendandi M, Inoges S, de Cerio AL-D, Pastor F (2015). 2-fluoro-RNA oligonucleotide CD40 targeted aptamers for the control of B lymphoma and bone-marrow aplasia. Biomaterials.

[136] Pastor F, Soldevilla MM, Villanueva H, Kolonias D, Inoges S, De Cerio AL (2013). CD28 aptamers as powerful immune response modulators. Mol Ther Nucleic Acids.

[137] Bai C, Gao S, Hu S, Liu X, Li H, Dong J (2020). Self-assembled multivalent aptamer nanoparticles with potential CAR-like characteristics could activate T cells and inhibit melanoma growth. Mol Ther Oncolytics.

[138] Takahashi M, Hashimoto Y, Nakamura Y (2022). Anti-TGF-β1 aptamer enhances therapeutic effect of tyrosine kinase inhibitor, gefitinib, on non-small cell lung cancer in xenograft model. Mol Ther Nucleic Acids.

[139] Tian H, Zhang T, Qin S, Huang Z, Zhou L, Shi J (2022). Enhancing the therapeutic efficacy of nanoparticles for cancer treatment using versatile targeted strategies. J Hematol Oncol.

[140] Pérez-Herrero E, Fernández-Medarde A (2015). Advanced targeted therapies in cancer: drug nanocarriers, the future of chemotherapy. Eur J Pharm Biopharm.

[141] Zhu L, Zhao J, Guo Z, Liu Y, Chen H, Chen Z (2021). Applications of aptamer-bound nanomaterials in cancer therapy. Biosensors (Basel)..

[142] Liu P, Ga L, Aodeng G, Wang Y, Ai J (2022). Aptamer-drug conjugates: new probes for imaging and targeted therapy. Biosens Bioelectron X.

[143] Mahmoudian F, Akbariqomi M, Heidari R, Ghahremani MH, Roshan N, Adabi M (2021). Designing a fluorescence padlock probe-based biosensor and colorimetric assay for the detection of G12D KRAS mutation. Biomark Med.

[144] Aljohani MM, Cialla-May D, Popp J, Chinnappan R, Al-Kattan K, Zourob M (2022). Aptamers: potential diagnostic and therapeutic agents for blood diseases. Molecules.

[145] Liu M, Wang L, Lo Y, Shiu SC-C, Kinghorn AB, Tanner JA (2022). Aptamer-enabled nanomaterials for therapeutics, drug targeting and imaging. Cells.

[146] Jabbari A, Sameiyan E, Yaghoobi E, Ramezani M, Alibolandi M, Abnous K (2023). Aptamer-based targeted delivery systems for cancer treatment using DNA origami and DNA nanostructures. Int J Pharm.

[147] Zavareh HS, Pourmadadi M, Moradi A, Yazdian F, Omidi M (2020). Chitosan/carbon quantum dot/aptamer complex as a potential anticancer drug delivery system towards the release of 5-fluorouracil. Int J Biol Macromol.

[148] Taghavi S, Ramezani M, Alibolandi M, Abnous K, Taghdisi SM (2017). Chitosan-modified PLGA nanoparticles tagged with 5TR1 aptamer for in vivo tumor-targeted drug delivery. Cancer Lett.

[149] Jalalian SH, Taghdisi SM, Shahidi Hamedani N, Kalat SA, Lavaee P, Zandkarimi M (2013). Epirubicin loaded super paramagnetic iron oxide nanoparticle-aptamer bioconjugate for combined colon cancer therapy and imaging in vivo. Eur J Pharm Sci.

[150] Moosavian SA, Abnous K, Akhtari J, Arabi L, Gholamzade Dewin A, Jafari M (2018). 5TR1 aptamer-PEGylated liposomal doxorubicin enhances cellular uptake and suppresses tumour growth by targeting MUC1 on the surface of cancer cells. Artif Cells Nanomed Biotechnol.

[151] Sayari E, Dinarvand M, Amini M, Azhdarzadeh M, Mollarazi E, Ghasemi Z (2014). MUC1 aptamer conjugated to chitosan nanoparticles, an efficient targeted carrier designed for anticancer SN38 delivery. Int J Pharm.

[152] Varnamkhasti BS, Hosseinzadeh H, Azhdarzadeh M, Vafaei SY, Esfandyari-Manesh M, Mirzaie ZH (2015). Protein corona hampers targeting potential of MUC1 aptamer functionalized SN-38 core–shell nanoparticles. Int J Pharm.

[153] Ghasemi Z, Dinarvand R, Mottaghitalab F, Esfandyari-Manesh M, Sayari E, Atyabi F (2015). Aptamer decorated hyaluronan/chitosan nanoparticles for targeted delivery of 5-fluorouracil to MUC1 overexpressing adenocarcinomas. Carbohyd Polym.

[154] Esfandyari-Manesh M, Mohammadi A, Atyabi F, Nabavi SM, Ebrahimi SM, Shahmoradi E (2016). Specific targeting delivery to MUC1 overexpressing tumors by albumin-chitosan nanoparticles conjugated to DNA aptamer. Int J Pharm.

[155] Jalalian SH, Ramezani M, Abnous K, Taghdisi SM (2018). Targeted co-delivery of epirubicin and NAS-24 aptamer to cancer cells using selenium nanoparticles for enhancing tumor response in vitro and in vivo. Cancer Lett.

[156] Abnous K, Danesh NM, Ramezani M, Lavaee P, Jalalian SH, Yazdian-Robati R (2017). A novel aptamer-based DNA diamond nanostructure for in vivo targeted delivery of epirubicin to cancer cells. RSC Adv.

[157] Guo X, Zhuang Q, Ji T, Zhang Y, Li C, Wang Y (2018). Multi-functionalized chitosan nanoparticles for enhanced chemotherapy in lung cancer. Carbohyd Polym.

[158] Alijani H, Noori A, Faridi N, Bathaie SZ, Mousavi MF (2020). Aptamer-functionalized Fe3O4@MOF nanocarrier for targeted drug delivery and fluorescence imaging of the triple-negative MDA-MB-231 breast cancer cells. J Solid State Chem.

[159] Zhang H-J, Zhao X, Chen L-J, Yang C-X, Yan X-P (2020). Dendrimer grafted persistent luminescent nanoplatform for aptamer guided tumor imaging and acid-responsive drug delivery. Talanta.

[160] Rață DM, Cadinoiu AN, Atanase LI, Bacaita SE, Mihalache C, Daraba O-M (2019). “In vitro” behaviour of aptamer-functionalized polymeric nanocapsules loaded with 5-fluorouracil for targeted therapy. Mater Sci Eng, C.

[161] Alizadeh L, Alizadeh E, Zarebkohan A, Ahmadi E, Rahmati-Yamchi M, Salehi R (2020). AS1411 aptamer-functionalized chitosan-silica nanoparticles for targeted delivery of epigallocatechin gallate to the SKOV-3 ovarian cancer cell lines. J Nanopart Res.

[162] Saravanakumar K, Sathiyaseelan A, Mariadoss AVA, Jeevithan E, Hu X, Shin S (2020). Dual stimuli-responsive release of aptamer AS1411 decorated erlotinib loaded chitosan nanoparticles for non-small-cell lung carcinoma therapy. Carbohyd Polym.

[163] Rata DM, Cadinoiu AN, Atanase LI, Popa M, Mihai C-T, Solcan C (2021). Topical formulations containing aptamer-functionalized nanocapsules loaded with 5-fluorouracil - an innovative concept for the skin cancer therapy. Mater Sci Eng, C.

[164] Wu J, Song C, Jiang C, Shen X, Qiao Q, Hu Y (2013). Nucleolin targeting AS1411 modified protein nanoparticle for antitumor drugs delivery. Mol Pharm.

[165] Yu Z, Li X, Duan J, Yang XD (2020). Targeted treatment of colon cancer with aptamer-guided albumin nanoparticles loaded with docetaxel. Int J Nanomed.

[166] Baneshi M, Dadfarnia S, Shabani AMH, Sabbagh SK, Haghgoo S, Bardania H (2019). A novel theranostic system of AS1411 aptamer-functionalized albumin nanoparticles loaded on iron oxide and gold nanoparticles for doxorubicin delivery. Int J Pharm.

[167] Xu L, Xu R, Saw PE, Wu J, Cheng SX, Xu X (2021). Nanoparticle-mediated inhibition of mitochondrial glutaminolysis to amplify oxidative stress for combination cancer therapy. Nano Lett.

[168] Xu L, He XY, Liu BY, Xu C, Ai SL, Zhuo RX (2018). Aptamer-functionalized albumin-based nanoparticles for targeted drug delivery. Colloids Surf B Biointerfaces.

[169] Khademi Z, Lavaee P, Ramezani M, Alibolandi M, Abnous K, Taghdisi SM (2020). Co-delivery of doxorubicin and aptamer against Forkhead box M1 using chitosan-gold nanoparticles coated with nucleolin aptamer for synergistic treatment of cancer cells. Carbohyd Polym.

[170] Duan T, Xu Z, Sun F, Wang Y, Zhang J, Luo C (2019). HPA aptamer functionalized paclitaxel-loaded PLGA nanoparticles for enhanced anticancer therapy through targeted effects and microenvironment modulation. Biomed Pharmacother.

[171] Yu MK, Kim D, Lee IH, So JS, Jeong YY, Jon S (2011). Image-guided prostate cancer therapy using aptamer-functionalized thermally cross-linked superparamagnetic iron oxide nanoparticles. Small.

[172] Niu W, Chen X, Tan W, Veige AS (2016). N-Heterocyclic Carbene-Gold (I) complexes conjugated to a leukemia-specific DNA aptamer for targeted drug delivery. Angew Chem Int Ed.

[173] Xie X, Li F, Zhang H, Lu Y, Lian S, Lin H (2016). EpCAM aptamer-functionalized mesoporous silica nanoparticles for efficient colon cancer cell-targeted drug delivery. Eur J Pharm Sci.

[174] Zhao J, Li D, Ma J, Yang H, Chen W, Cao Y (2021). Increasing the accumulation of aptamer AS1411 and verapamil conjugated silver nanoparticles in tumor cells to enhance the radiosensitivity of glioma. Nanotechnology.

[175] Li D, Zhao J, Ma J, Yang H, Zhang X, Cao Y (2022). GMT8 aptamer conjugated PEGylated Ag@Au core-shell nanoparticles as a novel radiosensitizer for targeted radiotherapy of glioma. Colloids Surf, B.

[176] He XY, Ren XH, Peng Y, Zhang JP, Ai SL, Liu BY (2020). Aptamer/Peptide-functionalized genome-editing system for effective immune restoration through reversal of PD-L1-mediated cancer immunosuppression. Adv Mater.

[177] Geng Z, Wang L, Liu K, Liu J, Tan W (2021). Enhancing anti-PD-1 immunotherapy by nanomicelles self-assembled from multivalent aptamer drug conjugates. Angew Chem Int Ed Engl.

[178] Yu L, Hu Y, Duan J, Yang X-D (2015). A novel approach of targeted immunotherapy against adenocarcinoma cells with nanoparticles modified by CD16 and MUC1 aptamers. J Nanomater.

[179] Lai X, Yao F, An Y, Li X, Yang X-D (2023). Novel nanotherapeutics for cancer immunotherapy by PD-L1-aptamer-functionalized and fexofenadine-loaded albumin nanoparticles. Molecules.

[180] Li W, Li F, Li T, Zhang W, Li B, Liu K (2023). Self-actuated biomimetic nanocomposites for photothermal therapy and PD-L1 immunosuppression. Front Chem.

[181] Camorani S, Tortorella S, Agnello L, Spanu C, d'Argenio A, Nilo R (2022). Aptamer-functionalized nanoparticles mediate PD-L1 siRNA delivery for effective gene silencing in triple-negative breast cancer cells. Pharmaceutics.

[182] Chang R, Li T, Fu Y, Chen Z, He Y, Sun X (2023). A PD-L1 targeting nanotheranostic for effective photoacoustic imaging guided photothermal-immunotherapy of tumor. J Mater Chem B.

[183] Yao F, An Y, Lai X, Li X, Yu Z, Yang XD (2023). Novel nanotherapeutics for cancer immunotherapy by CTLA-4 aptamer-functionalized albumin nanoparticle loaded with antihistamine. J Cancer Res Clin Oncol.

[184] Liu YJ, Dou XQ, Wang F, Zhang J, Wang XL, Xu GL (2017). IL-4Rα aptamer-liposome-CpG oligodeoxynucleotides suppress tumour growth by targeting the tumour microenvironment. J Drug Target.

[185] Yang X, Zhao J, Duan S, Hou X, Li X, Hu Z (2019). Enhanced cytotoxic T lymphocytes recruitment targeting tumor vasculatures by endoglin aptamer and IP-10 plasmid presenting liposome-based nanocarriers. Theranostics.

